# The dynamic response of Pine Island Glacier to two decades of intermittent ice shelf regrounding

**DOI:** 10.1038/s41467-026-75648-8

**Published:** 2026-07-29

**Authors:** Trystan Surawy-Stepney, Benjamin J. Wallis, Anna E. Hogg, Ian Joughin, Stephen L. Cornford, Pierre Dutrieux

**Affiliations:** 1 School of Earth, Environment and Sustainability, University of Leeds, Leeds, United Kingdom; 2 Polar Science Centre, Applied Physics Laboratory, University of Washington, Seattle, USA; 3 School of Geographical Sciences, University of Bristol, Bristol, United Kingdom; 4 British Antarctic Survey, Cambridge, United Kingdom

**Keywords:** Cryospheric science, Climate change

## Abstract

Over the last few decades, ice keels have intermittently bumped against the bedrock underneath Pine Island Ice Shelf. In general, it is thought that this process, termed regrounding, could enhance buttressing and potentially slow glacier flow or arrest retreat. Here, we test this hypothesis for Pine Island Glacier. Using offset tracking applied to satellite radar data, we reveal an extensive history of regrounding on the central shelf since 2009. Combining Earth observation and ice sheet modelling, we assess the direct effects this might have had on ice dynamics and indirect effects via its influencing on calving behaviour. Our results show that regrounding caused little additional drag on the base of the ice shelf. Furthermore, it plausibly contributed to the promotion of calving between 2015 and 2020 which is, in turn, held largely responsible for the increase in flow rate of the glacier over the last decade.

## Abstract (web summary)

Through satellite observations and numerical modelling, this study reveals intermittent regrounding beneath Pine Island Ice Shelf and shows it added little resistance to flow, but may have helped trigger rifting responsible for causing acceleration.

## Introduction

Pine Island Glacier (PIG) experiences one of the highest rates of mass loss in Antarctica^1^. As such, there has been significant interest over recent decades in documenting changes to its form and dynamics, particularly its grounding line location and flow speed[e.g.]^2–12^. Evidence from sediment cores suggests that the grounded ice stream previously extended to a sea-bed ridge ~40 km downstream of the current main grounding line before retreating in the mid-20th century^13–15^. The early stages of retreat from this ridge are difficult to characterise due to a lack of observations; however, satellite records show that by the 1990s retreat was ongoing and lasted until the late 2000s^2,6,7,14,16^. This retreat is thought to be due to ocean-induced thinning of the ice shelf, particularly near the grounding line^4^, perhaps aided by a lack of available stable grounding line positions^15,17^. The retreat was not entirely uniform across the glacier. Optical imagery shows that until the 1980s, the ice shelf remained pinned on a high point on the bathymetric ridge near its current centre^13^. Additionally, in the 1990s, a peninsula of grounded ice formed downstream of the current grounding line position, as uneven bathymetry caused retreat at the margins of the ice stream to outpace that in the centre^18^. Eventually, a subshelf cavity separated an island of grounded ice from the rest of the ice stream during the 2000s. Regions of grounded ice on the central shelf were observed throughout the early 2010s, suggesting the bumping of this thick column of ice along the subshelf bedrock as it was advected downstream^18^.

The flow speed of the glacier increased with each stage of grounding line retreat during this period[e.g.]^3,6,19,20^. By 2010, flow speed had risen to ~4000 m a^−1^ across the central ice shelf, an increase of ~1700 m a^−1^ from the measured 1974 speeds^21^. However, after reaching a narrow region of deep bed topography in the late 2000s, the grounding line appeared to stabilise and flow speeds plateaued correspondingly, perhaps signifying that the pattern of retreat and acceleration that had dominated the preceding 50 years had come to an end. In 2017, however, the speed began to increase again^12^ with our records showing an increase of ~1000m a^−1^ at the grounding line between 2017 and 2021. This increase in speed has been attributed^12^ to the four large calving events between 2015 and 2020 that more than doubled the steady state calving rate, leading to a 20 km retreat in the terminus position. Other factors, such as degradation in the southern shear margin have been implicated as well^22,23^. The rifts responsible for these calving events started in the central ice shelf before propagating towards the shear margins and detaching large tabular icebergs. This marked a departure from the typical pattern of rift propagation inward from shear margins or lateral boundaries. It has been suggested that, in part, this was due to a change in direction of flow of the terminus region of the ice shelf as the northern shear margin retreated^8^.

A glacier undergoing acceleration and grounding line retreat, as PIG has, can lead to an ice shelf with highly heterogeneous thickness. As the grounding line migrates, the thickness of ice crossing it changes. Additionally, because melt rates depend on ice thickness, flow speed, and evolving ocean conditions, different parts of the ice shelf experience different cumulative basal melt over time. This is seen, for example, in the thick column of ice that was advected downstream after the retreat of the peninsula of grounded ice in the 2000s^18,24^. Similarly, the grounding line geometry of PIG, and ocean conditions at the time at which ice flows over the grounding line, have been observed to leave an imprint on the thickness that can persist for decades^25^. In general, one might assume that localised, positive perturbations in the thickness of an ice shelf could have the effect of slowing down a marine-terminating glacier, or preventing its grounding line from retreating^26^. This could be due to thicker ice increasing drag at the lateral margins of the ice shelf, or by causing parts of the ice shelf to come into contact with subshelf bedrock causing resistance to the flow. This article is concerned with the latter point. Firstly, we ask how much regrounding has occurred on Pine Island’s central ice shelf over the last 15 years and, secondly, what impact this had on the dynamics.

## Results

### A timeline of intermittent regrounding

We have constructed a record of regrounding on the central Pine Island ice shelf from 2009–2024 using differential-range offset tracking (DROT) methods^18,27^. DROT involves assessing the temporal variability of displacements measured in the range direction by an offset-tracking procedure applied to synthetic aperture radar (SAR) data (in our case, TerraSAR-X or Sentinel-1 data). For floating ice, we expect a large component of the variability in the range-direction offsets to be due to the vertical motion of the surface over tidal cycles, and for this to be absent in those measured over grounded ice.

**Fig. 1:**
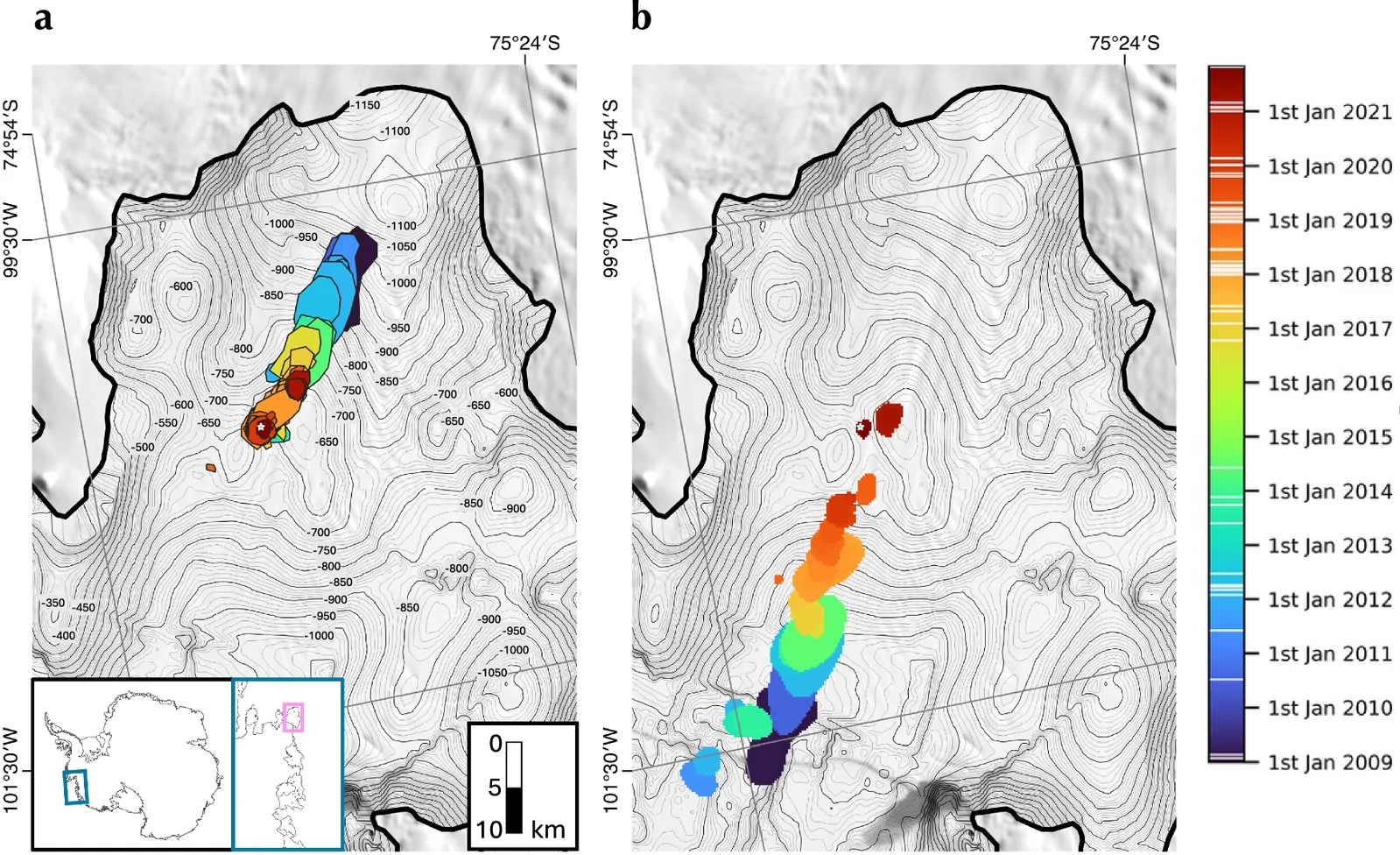
Regrounding on Pine Island Ice Shelf between 2009 and 2022. **a** Coloured areas show the locations and times at which regrounding occurred. **b** Coloured areas show the areas of the ice shelf that regrounded remapped to a common date in September 2021 - assuming no calving. A small white star in the location of the regrounding in September 2021 indicates the inferred location of the bathymetric peak. Contouring shows the suggested sub-shelf bathymetry^30^. The solid black line shows the main grounding line in 2011^69^. The background image on the grounded ice is the MODIS Mosaic of Antarctica^37^.

Figure 1 shows the pattern of regrounding we observe between January 2009 and 2024 on Pine Island Ice Shelf. This appears consistent with the findings of an independent study published concurrently with this work^28^. Regrounding has occurred intermittently in a narrow strip in the centre of the ice shelf (Fig. 1a, coloured areas), on bedrock sloping upward toward the bathymetric peak that formed a prominent ice rumple in the 1980s^13^. Bathymetry is based on gravimetry inversion^29^, supplemented with Autosub observations^30^. We infer the location of the bedrock high-point to be the centre of the region over which ice was observed to most frequently ground (Fig. 1, white star). To reconstruct the regrounding history, we used satellite-derived, time-evolving ice-flow velocity fields to remap grounded regions through time, tracking individual ice columns from where they grounded to their positions at the end of 2021 (Fig. 1b, coloured areas). This reveals a complex history of contact between the ice shelf and bed, rather than the repeated regrounding of a single keel. No regrounding is detected after 2021, and the grounded area appears to have shrunk between 2015 and 2021, possibly reflecting thinning of the shelf—typically several metres per year over the central shelf^31^—or an end to the advection of a thick parcel of ice over the bathymetric high-point. Subsequent regrounding could still occur undetected by this method, for example where the grounding persists for less than a full tidal cycle. This might be recovered by differential SAR interferometry (D-InSAR) which would exhibit greater sensitivity to short-lived, tidally driven flexure. Hence, we assume some regrounding is likely to have occurred since 2021. Observations produced using D-InSAR go back to the early 1990s^32^ and show grounded spots coincident with those we observe in 2009 and 2011, but none prior to this save a small spot in 1999. Even so, we cannot be confident that 2009 was the start of regrounding on the central shelf as the observations are sparse (even small reductions in interferometric coherence could degrade the observations to a point at which light grounding - such as that which persists for only part of the tidal cycle—is not easily visible). Thus, one could imagine the train of coloured areas in Fig. 1 extending far downstream of the horizon of our observations.

The DROT method requires high-quality measurements of range offsets to recognise grounded areas. Due to a variety of environmental factors, this is not consistently possible throughout the observational period. Hence, having no measurement of regrounding does not necessarily mean that the ice shelf was not grounded. Note, for example, that most of our observations of regrounding appear between 2017 and 2021, where the Sentinel-1 satellites had their most consistent orbit geometries and shortest repeat period (6 days). However, the conclusion that regrounding occurred throughout the observational period with regularity is robust.

### Regrounding contributed no noticeable drag

The most direct way in which the regrounding of the central ice shelf could have influenced Pine Island Glacier’s dynamics is by altering the horizontal stress balance through additional basal friction or form drag within the ice, or vertical shear within a deformable bed. To test this, we solved a series of inverse problems using the BISICLES ice sheet model^33^ aimed at extracting any signal in the record of ice speed data that might indicate areas of non-zero basal friction. We focused on the period 2016–2021, when our observations indicate the most sustained regrounding (Fig. 1). For each month in this period, we artificially grounded the whole ice shelf by raising the elevation of the bedrock to contact the ice base and then solved the inverse problem for basal friction using 200 m resolution, monthly-averaged ice speed mosaics^34^. This speed data is independent of that used in the DROT method. We updated calving-front positions after major calving events (2015, 2017, 2018, 2020) to ensure appropriate driving stresses. Our focus is not on the magnitude of the inferred basal stresses, which are only notional given that the whole ice shelf is artificially grounded, but on whether they reveal any spatial preference for higher stickiness near the bathymetric high point or any temporal variability coinciding with observed regrounding events.

**Fig. 2:**
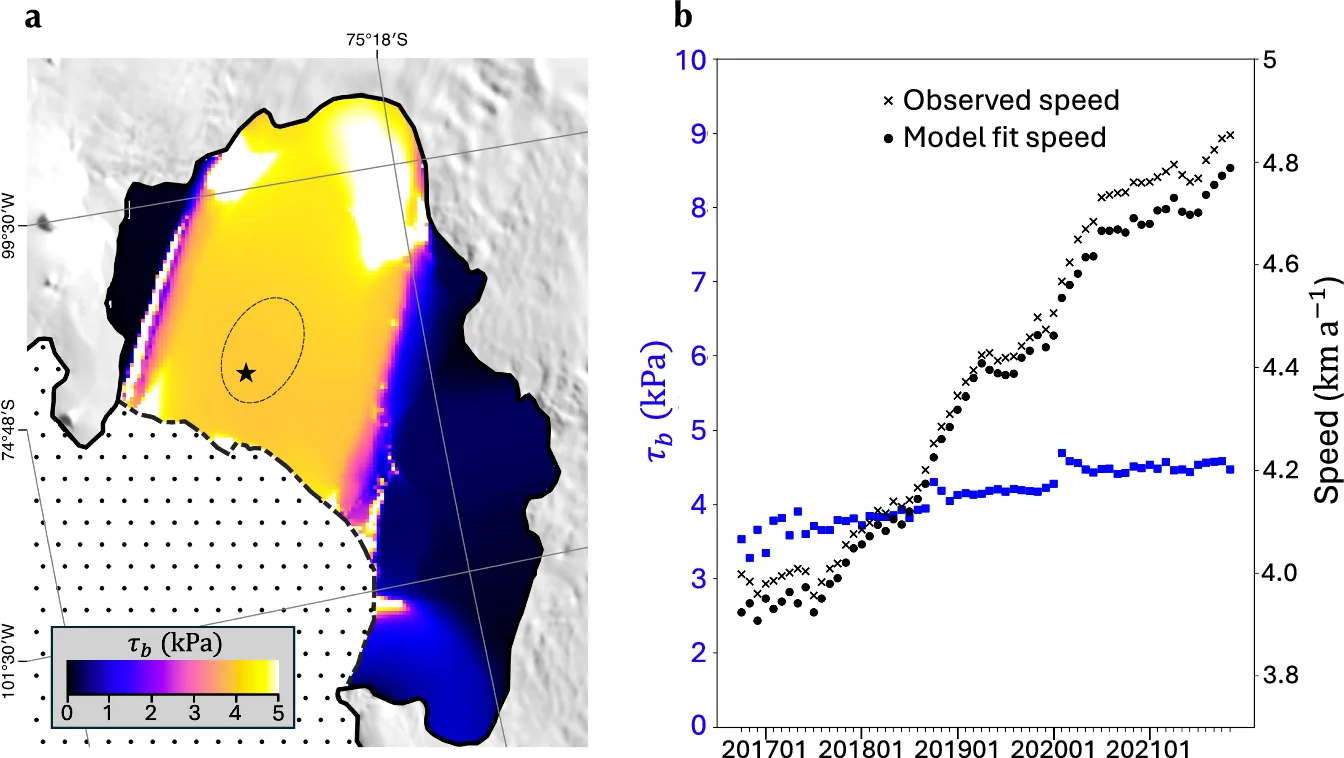
Results of the inverse problem for basal friction over the artificially-grounded ice shelf between 2016 and 2021. **a** Mean basal friction recovered over the full time series of inverse problems. The oval indicates the region in which regrounding frequently occurred, from which the time series in (**b**) are extracted. The black star indicates the inferred location of the bathymetric peak. **b** Time series of mean basal friction recovered by the inverse problem, mean observed ice speed and mean ice speed after convergence of the inverse problem over the region shown by the black dashed line in (**a**). The calving front in (**a**) is that of January 2020. The solid black line is the 2011 grounding line^69^. The background image on grounded ice is the MODIS Mosaic of Antarctica^37^. The dotted region indicates open ocean.

The results of the inverse problem show low basal stresses across the artificially grounded ice shelf, averaging around 4 kPa throughout the period 2016–2021 (Fig. 2). For context, we take 100 kPa as a representative stress scale - roughly the difference in vertically averaged hydrostatic pressure between ice and seawater at the calving front for ice thicker than around 200 m. Although the solution to the inverse problem indicates non-zero basal stresses, these do not reflect real traction; they are a reflection of the ill-posed nature of the inverse problem, which distributes small fictitious stresses across the shelf to minimise velocity misfit. This artefact is offset by lower effective viscosities inferred in the shear margins. While the recovered stress field does exhibit some spatial variability (Fig. 2a), it is smooth and lacks any localisation near the bathymetric high point where regrounding was observed (Fig. 1a). Similarly, the time series of mean basal stress over the grounded region (Fig. 2b) shows fluctuations, but these do not coincide with periods of regrounding identified in the satellite record. These results show intermittent regrounding left little discernible imprint on the velocity field.

**Fig. 3:**
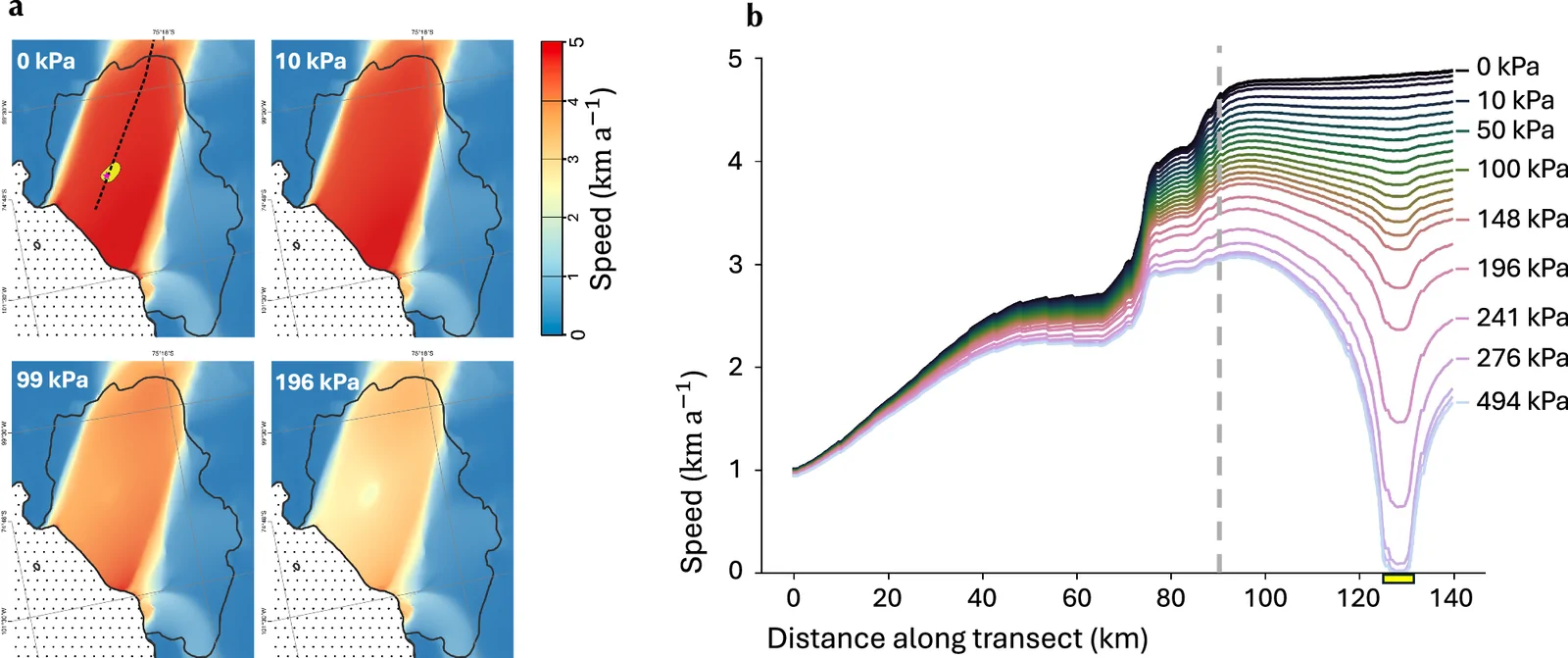
The effect of non-zero basal stress on the speed of the ice shelf in May 2018. **a** Ice speed given different levels of stickiness applied over the region shown in yellow in the top-left panel. The stickiness coefficients resulted in basal tractions of 0, 10, 99, 196 kPa. The solid black line shows the 2011 grounding line^69^. The dot-dashed black line shows the calving front in May 2018. **b** Profiles of ice speed along the black dashed line shown in the top-left panel of (**a**) for 25 different stickiness coefficients. Selected basal tractions are shown next to some of the lines. The grey dashed line indicates the location of the grounding line shown in (**a**) and the yellow box under the x-axis shows where the transect crosses the region of imposed grounding.

To constrain the sensitivity of the ice speed to the introduction of isolated regions of non-zero basal stress, we carried out further diagnostic simulations with different degrees of stickiness prescribed in the region of the bathymetric peak, assuming the ice shelf was initially ungrounded. We initialised the model in a geometry reflecting Pine Island’s configuration in May 2018 (with the ice shelf fully floating), using speed data from that month^34^. We then artificially grounded a small part of the central shelf reflecting the grounded area in mid 2018 (Fig. 3a, yellow shape) and progressively increased the stickiness over that region, each time recalculating the ice speed. The artificial grounding was done by locally raising the height of the subshelf bathymetry over the small region we wanted to ground. We assumed a regularised Coulomb sliding law with a regularisation speed of 300m a^−1^^35,36^ - such that stresses are effectively plastic at the flow speeds exhibited by Pine Island’s ice shelf. Figure 3 shows that the introduction of comparatively small basal stresses in the region has a large effect on the ice speed. For example, the addition of 10 kPa of basal stress over the area shown in Fig. 2a reduces the speed in the region by ≈250 m a^−1^) (over 5%). However, the timeseries of observed ice speeds (Fig. 2b - crosses) show monthly variations in ice speed do not reach this magnitude. Hence, we consider 10 kPa to be an upper bound to the basal stress induced by regrounding. We conclude from both sets of modelling experiments detailed in this section that the direct impact of regrounding on the dynamics of Pine Island Ice Shelf through the addition of basal friction was minimal.

### Regrounding and the development of rifts

In addition to the direct impact that regrounding could have had on glacier dynamics by supplying basal friction, it has been previously noted that it could have also influenced rift formation and, by extension, calving^12^. This would constitute an indirect pathway to influencing dynamics. As discussed above, the major increase in speed on PIG in the 2010s has been largely attributed to the unusually high calving rate between 2015 and 2020 in which four large tabular icebergs detached, compared to a rate of around one per decade previously^8,12^. Each of these four calving events followed a similar pattern: rifts initiated near the centre of the shelf and propagated laterally toward the margins, producing large tabular icebergs that sometimes disintegrated soon after. This contrasts with previous tabular calving on PIG, which was associated with rift initiation at ice-shelf margins^8^. The causes of this change in calving style remain unclear, especially as this elevated calving rate appears to have subsided, with no major calving events since 2020.

Several factors likely increased the susceptibility of the ice shelf to calving. The terminus of Pine Island Ice Shelf has changed shape considerably since the 2000s with a particularly large retreat on its northern side since 2007^8,37^. Additionally, both the southern and northern shear margins have weakened and retreated^22,38^—a pattern of change that has been ongoing since the 1990s^39^. These changes will have acted to redistribute resistive stresses across the self. Notably, translating shear stresses formerly localised in the shear margins into longitudinal stress in the central ice shelf. This background increase in longitudinal stress will have, in turn, acted to increase the probability of rift formation. Evidence for this is given by the fact that calving occurred in-line with the seaward extent of the well-consolidated parts of the shear margins. However, this redistribution of internal stress does not explain such a dramatic increase in calving rate, the manner of calving, with rifts propagating laterally from the centre, and, most notably, why calving reduced after 2020 given that the shear margins have not regrown. These facts suggest the seeding of weaknesses in the central shelf by means other than increased internal stresses caused by the retreating shear margins. Our observations show a conspicuous spatial relationship between the location of cracks in the central shelf and the areas of ice known to have grounded.

**Fig. 4:**
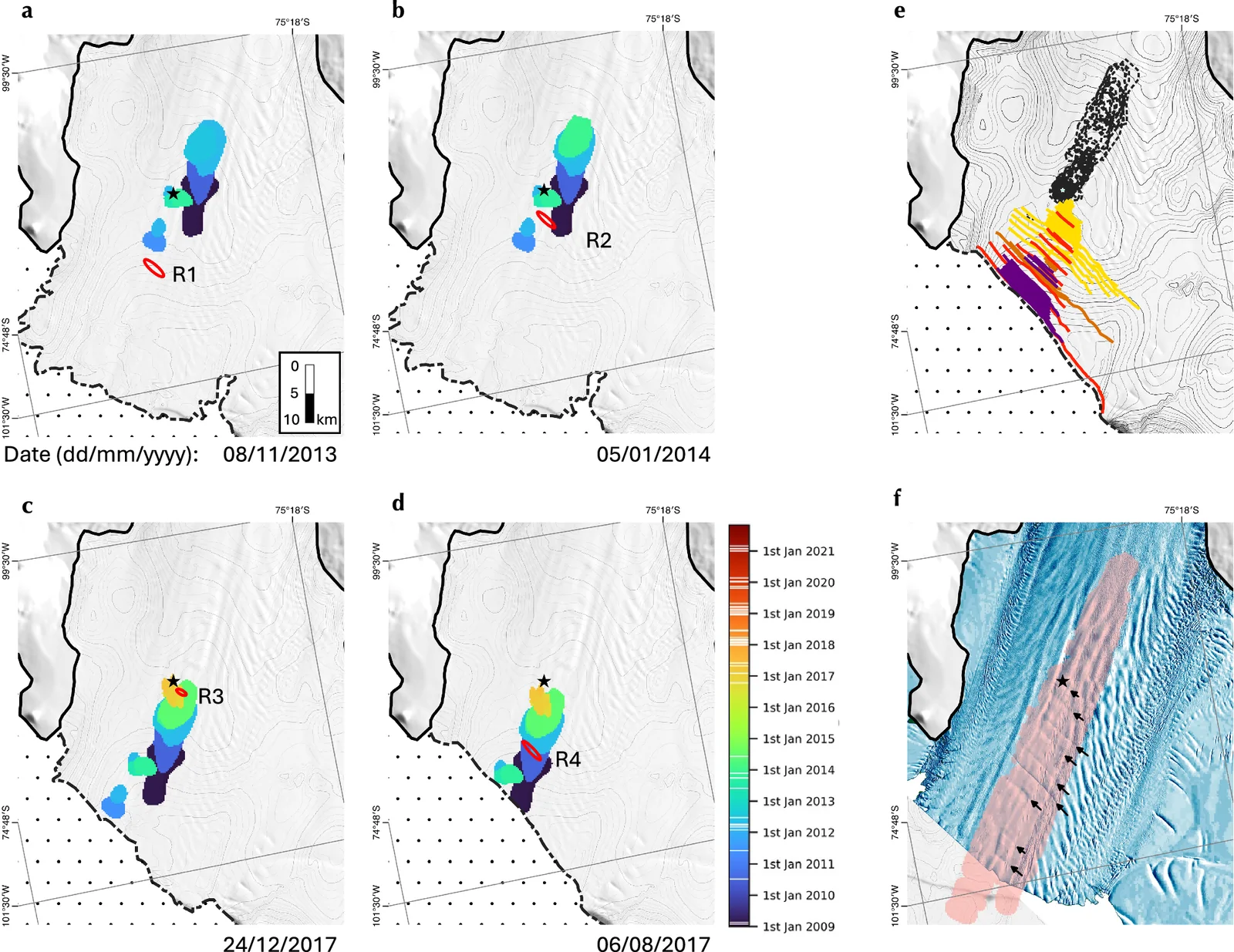
**a**–**d** Each panel shows, with a red ellipse, the location at which each rift that led to a calving event between 2015 and 2020 was first observed. The date corresponding to this observation is shown below the panel. Each panel also shows the columns of ice that were observed to have grounded coloured by the date at which grounding occurred, remapped to the date shown below each panel. **e** The evolution of each rift R1-4 from satellite imagery traced from the first image in which they were visible to the image before the detachment of an iceberg. Black dashed lines show where we detected regrounding (the boundaries of the regions shown in Fig. 1a). **f** Landsat-8 OLI image from November 2014 with surface depressions corresponding to crevasses or rifts highlighted with black arrows. The pink shaded region shows the lateral boundaries within which parts of the ice shelf were observed to have regrounded. The black star in (**a**–**d**) and (**f**), and the white star in (**e**) show the inferred location of the bathymetric peak, as shown in the other figures. The solid black lines show the main grounding line in 2011^69^. The dot-dash line in (**a**–**e**) shows the approximate calving front location. The background image for (**a**–**e**) is the MODIS Mosaic of Antarctica^37^. Contouring shows the sub-shelf bathymetry^30^.

Using Sentinel-1 SAR imagery (resolution: 5 × 20 m posted to a 10 × 10 m grid), augmented by Sentinel-2 (resolution: 10 × 10 m) and Landsat-8 (resolution: 30 × 30 m) optical imagery, we tracked the rifts that led to the four major calving events to as close to their nascency as possible—where they became too difficult to see or when data ceased to be available. We refer to the rifts that led to the large calving events in August 2015, September 2017, October 2018 and January 2020 as R1, R2, R3, and R4, respectively. We tracked the parts of the ice shelf that regrounded by remapping masks of grounded ice between timesteps using a time-varying velocity field, as with Fig. 1b. Our observations show that early-stage rifts consistently appeared downstream of the bathymetric high-point, on which the ice frequently regrounded, and propagated laterally outwards from there (Fig. 4a–e). In each case, the rifts appeared within a few kilometres of regions that had previously regrounded (Fig. 4a–d). This is close enough to lie within the hinge zones that surrounded the grounded regions^40,41^, i.e. areas in which, under tidal motion, ice is neither supported fully at the base by either bedrock or hydrostatic water pressure, and where additional stresses can be felt above the background (Sec. 2.3.1). Looking at a fuller picture of crevassing on the central shelf, to a 2014 Landsat-8 image^8^ we find that all visible surface depressions corresponding to cracks in the ice (Fig. 4f, black arrows) appear downstream of the bathymetric high-point, within the lateral boundaries within which we observed grounded ice (Fig. 4f, pink stripe). Though far from definitive, these observations hint that regrounding was important in either seeding crevasses in the ice, or further developing pre-existing features.

#### An estimate of the stresses induced by tidal hinging

Grounding in the centre of an ice shelf can influence fracture in several ways. One possibility is that grounding forces the ice to bend over sharp bed features, creating vertical shear stresses, but we see no evidence for this in satellite imagery of the surface. Another is that basal traction redistributes horizontal viscous stress within the rest of the shelf. For example, transferring shear stresses from the shear margins to longitudinal stresses just downstream of the grounded area. This can be seen, for example, in the higher ice speed gradients exhibited downstream of the enforced grounding shown in Fig. 3b as traction is increased. However, our modelling shows that basal stresses during regrounding were small, so we consider this unlikely. Finally, regrounding could promote fracture through tidal hinging.

**Fig. 5:**
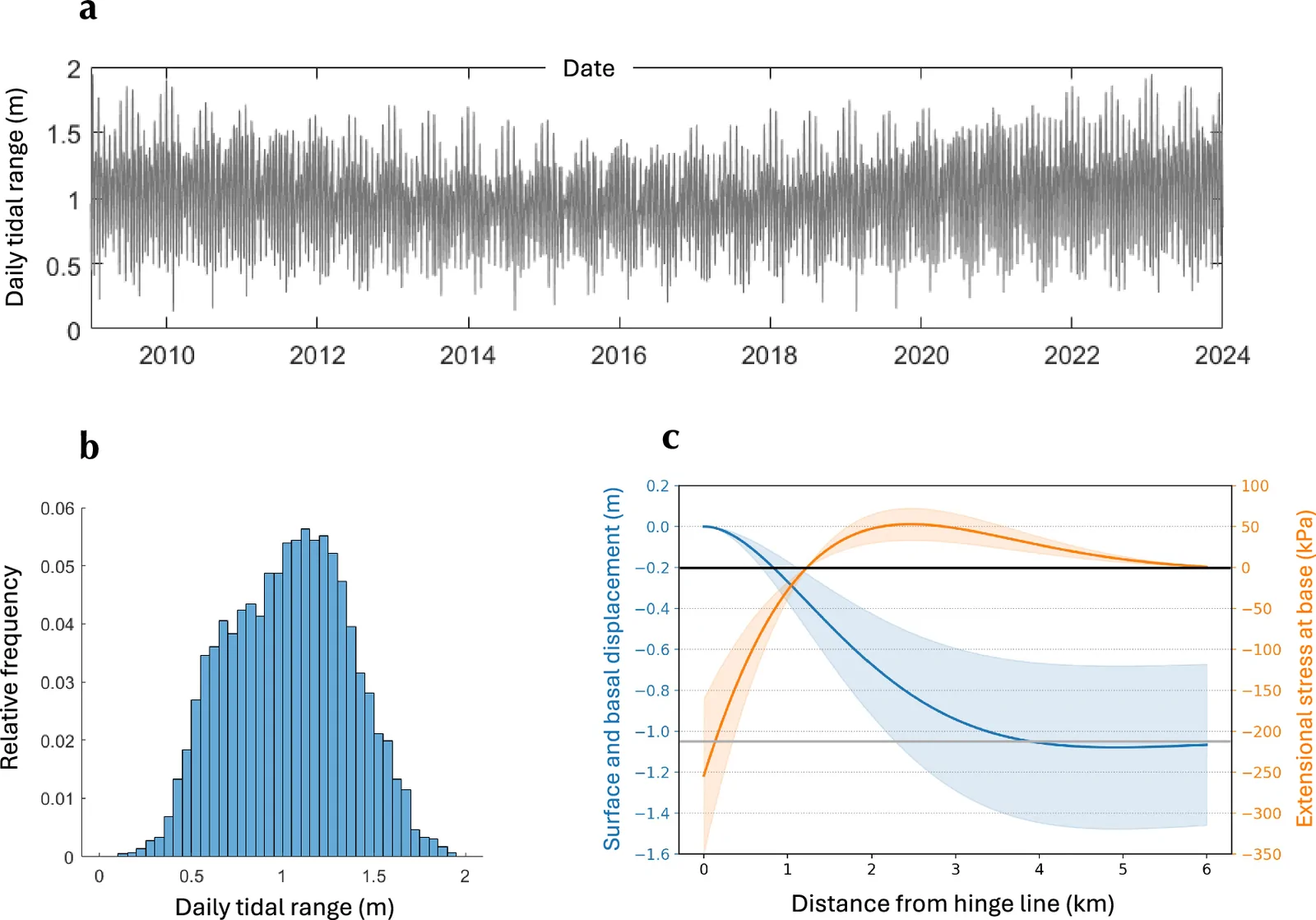
Tidally induced stress in a one dimensional elastic beam anchored at the bathymetric high-point. **a** A timeseries of daily tidal ranges in the location of the bathymetric high point from the CATS2008 tide model^42^. **b** A histogram of daily tidal range in the location of the bathymetric high point from 2009–2024. **c** The deflection (blue line) and induced stress at the bottom surface of a one-dimensional elastic beam subject to tidal range of mean and standard deviation shown in (**b**). Parameters used to calculate deflection and stress are given in the main text.

Using a simple, one-dimensional, elastic beam formulation^40^, we assessed the potential increase in longitudinal stress placed on the ice due to tidal flexure. We assumed a Poisson’s ratio of *ν* = 0.3, and a Young’s modulus of *E* = 1 GPa for the ice, and a constant thickness of *h* = 550 m—corresponding to the average thickness over the yellow region shown in Fig. 3a. Between 2014 and 2024, the daily tidal range in the location of the bathymetric high-point averaged 1.04 ± 0.38 m (Fig. 5a, b) according to the CATS2008 tide model^42^. Under the assumptions made, this would have induced a tensile stress of 209.3 ± 78.9 kPa at the surface of the ice at the boundary of the grounded regions (Fig. 5c). Under this model, flexure would have also induced a maximum tensile stress at the base of the ice *x* = 2.5 km from the hinge line, ranging between 43.5 ± 16.4 kPa in magnitude (Fig. 5c). These values are of comparable magnitude to background viscous stresses and suggested values for the fracture toughnesses of glacier ice [e.g.]^43^. Hence this explanation as to why rifts consistently appeared downstream of the bathymetric high point where grounding occurred at least passes the test of plausibility.

## Discussion

### Why was basal friction so low?

Our modelling results show that contact between the ice base and the bathymetric high point was unlikely to have introduced basal stresses above around 10 kPa. This result suggests that regrounding occurred on weakly deformable till, or that a strong hydrological connection to the ocean was maintained throughout regrounding, resulting in low effective pressures at the base of the ice. The lack of discernable surface expression of the regrounding in satellite imagery also indicates a low height above floatation in the grounded regions. One implication of this result is that it is unwise to extrapolate basal stickiness coefficients to sub-shelf cavities when running simulations of marine ice sheets in which regrounding can occur, as this could lead to over-estimation of basal traction. It also, once more, serves to highlight the merits of effective-pressure-dependent sliding laws which might help account for low traction when ice regrounds on the sea floor.

### Could tidal hinging promote fracture?

We showed that, under an elastic assumption, tidal flexure around grounded spots could have generated longitudinal extensional stresses on the order of 100 kPa at the surface and 50 kPa at the base. A more complete account would be given by treating the ice viscoelastically^44^. In such a case, viscous processes such as creep and recrystallisation near crack tips would reduce the stress available to produce new crack surface area. However, even in this case it is plausible that tidal motion could drive subcritical fatigue failure due to repeated cyclic loading. It is typical to think of ice as a material that fails brittlely and super-critically, though it has been shown to be subject to fatigue failure on short timescales (e.g.^45^), although this study pertains to freshwater ice rather than glacier ice. However, future laboratory work should aim to investigate such processes on ice for loading occurring on Maxwell timescales.

Though the observations are suggestive and there are options for a mechanistic explanation, there remain challenges to the idea that regrounding seeded crevasses on the central shelf. For example, no rifts formed in areas that grounded after 2017, and our satellite images of the surface are insufficient to judge whether cracks formed at the time of regrounding. In addition, it is well-known that basal crevasses can persist for decades before producing a clear surface expression[e.g.]^46^. Hence, it is plausible that the features shown in Fig. 4f originated far upstream of the bathymetric high-point, e.g. at the grounding line during a period of retreat, and became visible downstream of it only coincidentally. It is also possible that ocean conditions were at different times more conducive to focussed channelised melting in pre-existing basal crevasses, increasing their depth. Thus, while tidal hinging is a candidate mechanism, it is not the only possibility.

### Implications beyond Pine Island Glacier

The major result of this article is that regrounding provided little resistance to the flow of Pine Island Ice Shelf (under 10 kPa of basal stress). This is perhaps surprising given the large surface areas of the regrounded regions (Fig. 1). Intriguingly, our results suggest that regrounding may also have contributed to seeding or opening the fractures in the central shelf implicated in the elevated calving that caused the dynamic change experienced by the ice shelf over the last decade. Taken together, these conclusions have two particular implications of note beyond understanding the story of Pine Island Glacier. Firstly, it is generally accepted that most calving occurs as a result of englacial stresses associated with viscous dynamics^47,48^. However, many numerical models will not be able to account for additional stress due to tidal flexure, never mind the viscoelastic fracture physics required to predict failure. Hence, should the rifts have truly developed in part due to tidal flexure, existing models might find it difficult to replicate the dynamic changes that have occurred on PIG over recent decades without considerable development. Secondly, these results have implications for the general way in which we conceptualise regrounding in the context of a marine ice sheet in retreat. It is sometimes assumed that regrounding should have a stabilising effect, however, the example here shows that intermittent grounding coincided with increased flow speed and degradation of the remaining ice shelf, implying that its effect can be negligible or even counterproductive.

## Methods

### Identifying grounded spots using differential-range offset tracking

**Fig. 6:**
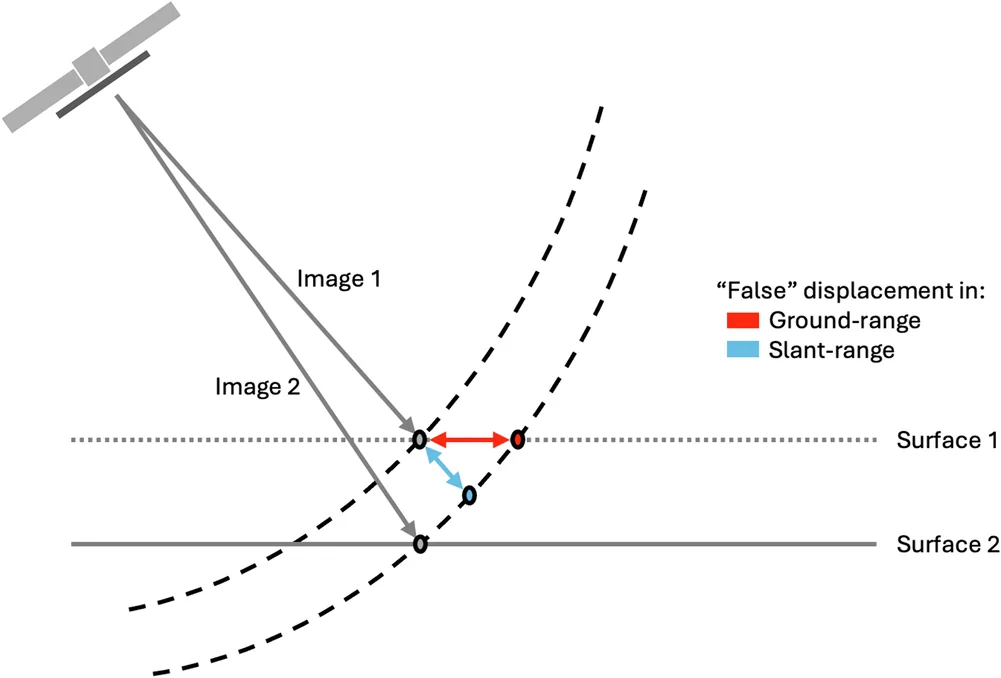
The source of error in range-displacements generated by SAR offset tracking over surfaces with varying vertical location. Vertical displacements of the surface (unaccounted for in the coregistration of backscatter data) between image acquisitions 1 and 2 are interpreted as ground-range displacements (labelled “false displacement'').

The displacement maps used to generate data on glacier flow speed are often generated from synthetic aperture radar data using offset tracking procedures^49^. In this method, coregistered backscatter images are cross-correlated in overlapping patches, allowing one to read off the displacement of features between the image acquisitions. To coregister the images, a model of the scattering surface of is required. If this model fails to account for vertical motion of the surface between the image acquisitions, as in our case, there will be an error in the location of features in the range direction (either slant-range, or ground-range, depending on the coordinates used when the cross correlation is performed). Figure 6 shows how the vertical motion between the surface appears as a false displacement in the ground- or slant-range direction. This leads to higher variances in the range displacements measured over floating ice compared to grounded ice, as these are subject to vertical tidal motion^18^.

We generated ground-range displacement maps over Pine Island Ice Shelf from September 2014 onwards using all available Sentinel-1 acquisition pairs separated by 6 or 12 days. To remove the background range displacement field, we subtracted the median range displacement field for a 6-month window centred on the mid-date of each ice speed map, leaving only the ground-range displacement anomaly due to tidal motion of the ice shelf. This approach differs from a simple double-difference of temporally adjacent ground range displacement maps, but by subtracting the median, we increase the volume of high-quality tidal displacement measurements, because we do not require two consecutive high-quality tracking results. Using the CATS2008 tide model^42^, we filtered these by removing displacement data from acquisitions with differential tidal amplitude below 0.5 m. In total, this produced 183 displacement maps. Additionally, we used an additional 57 displacement maps derived from TerraSAR-X over the period 2007^18^. We inspected this time series of range displacement anomaly maps (this time in slant-range, rather than ground-range coordinates) to identify regions of temporary grounding in the central PIG shelf. These appear as spots of low slant-range displacement anomaly that match the values in areas of known grounded ice (inland of the main grounding line). Areas of grounding in the central shelf were then delineated using GIS software, to form a time series.

**Fig. 7:**
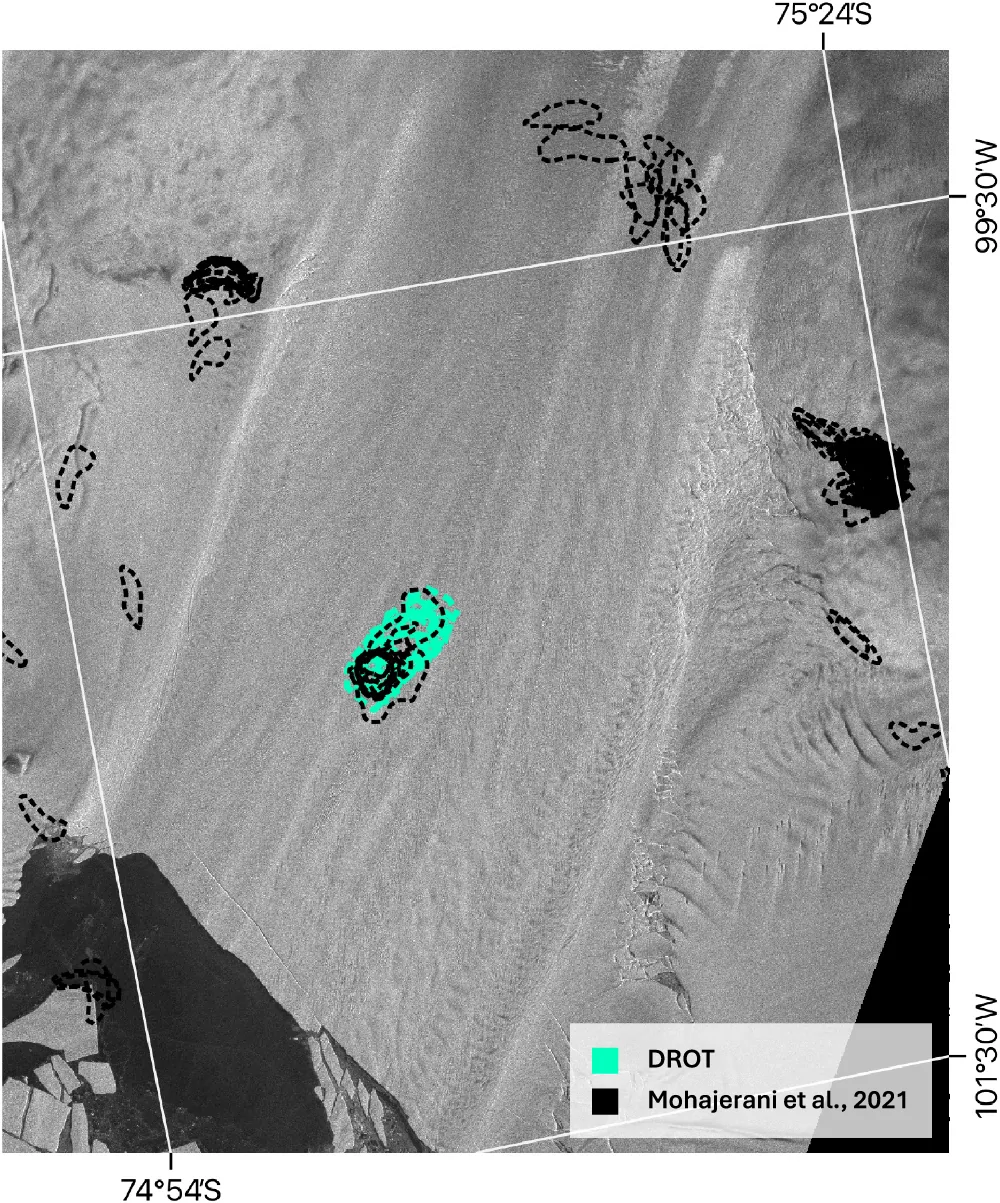
A comparison between our observations of central-shelf grounding in 2018 (cyan) to D-InSAR-derived observations^50^ (black). The data are overlain on a Sentinel-1 backscatter image of Pine Island Glacier from June 2018.

Often, differential SAR interferometry is used to locate grounding lines (e.g.^32^). By differencing interferograms, regions of tidal flexure can be identified and their inland limit delineated. We chose to use DROT rather than D-InSAR for this study as we prioritised dense temporal coverage in our observations of regrounding. DROT requires a lower level of interferometric coherence between image acquisitions, which is often limited in this part of Antarctica due to snow deposition and changes in surface moisture. In order to understand the consistency of the DROT and D-InSAR approaches, we compared our observations of grounded locations in 2018 to those generated using a deep-learning-based approach for extracting grounding lines from maps of interferometric fringes and coherence data^50^. Figure 7 shows good agreement between the D-InSAR-derived grounded regions and our own on the central shelf, in both location and size. Interestingly, they find a large number of grounded spots near the main grounding line, perhaps due to the greater sensitivity of the InSAR approach over DROT.

### Inverse modelling of basal stress

To assess whether regrounding introduced measurable basal traction, we performed a series of inverse problems using the BISICLES ice sheet model^33^, in which we pretend that the ice shelf is fully grounded. These were designed to infer spatial and temporal variations in basal stress over the central shelf during periods of observed regrounding. BISICLES is an adaptive mesh model, and we use nested meshes with a highest resolution of 500 m on floating ice and at the grounding line and up to 8 km inland of the grounding line.

We focussed on the period 2016–2021, during which our measurements show the most sustained regrounding. For each month, we artificially raised the bedrock beneath the entire ice shelf to enforce grounding, thereby allowing the model to infer basal friction coefficients (*C*) over regions that are known to be floating in reality. This tests whether the observed velocity fields contain any signal indicative of basal traction in the vicinity of the bathymetric high point, or basal traction that fluctuates in time. In order not to modify driving stresses, the bedrock was raised to fit the base of the ice shelf, such that the thickness and surface slope of the ice shelf remained the same.

The inverse problem is an optimisation problem solved for the basal traction coefficient *C* and the ice stiffness *ϕ* for each month of data. It aims to minimise a cost functional comprising a misfit term measuring the difference between modelled and observed surface velocities, and regularisation terms on *C* and *ϕ*, both in space and time. This can be written:

$$({C}_{i},{\phi }_{i})=	\arg {{\min }_{{C}_{i},{\phi }_{i}}}\{{{{{\mathcal{J}}}}}_{m}({u}_{i},{u}_{{o}_{i}})+{\alpha }_{C}{{{\mathcal{J}}}}({C}_{i})+{\alpha }_{\phi }{{{\mathcal{J}}}}({\phi }_{i})+\frac{{\alpha }_{C}^{{\prime} }}{\Delta t}{{{{\mathcal{J}}}}}^{{\prime} }({C}_{i},{C}_{i-1})+\frac{{\alpha }_{\phi }^{{\prime} }}{\Delta t}{{{{\mathcal{J}}}}}^{{\prime} }({\phi }_{i},{\phi }_{i-1})\},\\ 	{{{\rm{such}}}}\,{{{\rm{that}}}}\,G({u}_{i},{h}_{i},{C}_{i},{\phi }_{i})=0$$

where the indices *i* indicate the timestep, *C* indicates the basal stickiness coefficient that is of principal interest to us, *ϕ* is the ice stiffness, *u* is the modelled speed, *u*_o_ is the observed ice speed, $\{{\alpha }_{C},{\alpha }_{C}^{{\prime} }\,{\alpha }_{\phi },{\alpha }_{\phi }^{{\prime} }\}$ are regularisation coefficients, ${{{{\mathcal{J}}}}}_{m}$ is the misfit functional, $\{{{{\mathcal{J}}}},{{{{\mathcal{J}}}}}^{{\prime} }\}$ are regularisation functionals and *G*(*u*_*i*_, *h*_*i*_, *C*_*i*_, *ϕ*_*i*_) = 0 are the momentum-balance equations. Here, we chose these to be the vertically integrated, first-order approximation to the Stokes equations called the Shallow Stream Approximation:

1 $$G({{{\bf{u}}}},h,C,\phi )={{{\boldsymbol{\nabla }}}}\cdot [\phi h\bar{\eta }({{{\boldsymbol{\nabla }}}}{{{\bf{u}}}}+{({{{\boldsymbol{\nabla }}}}{{{\bf{u}}}})}^{\top }+2({{{\boldsymbol{\nabla }}}}\cdot {{{\bf{u}}}}){{{\mathcal{I}}}})]-Cf({{{\bf{u}}}})-{\rho }_{i}gh{{{\boldsymbol{\nabla }}}}s,$$

where ${{{\bf{u}}}}={({u}_{x},{u}_{y})}^{\top }$ is the horizontal velocity, $\bar{\eta }$ is the vertically-integrated effective ice viscosity, *ρ*_*i*_ is the density of ice, *h* is the ice thickness and *s* is the ice surface. Given that we are interested in the basal stress rather than the coefficient *C*, the choice of the functional form for the sliding law *f*(**u**) is arbitrary. Hence, we used a linear form *f*(**u**) = **u** for ease of computing adjoint sensitivities.

We used monthly velocity mosaics from ENVEO^34^ as input data, which is independent of the data used to identify grounded parts of the shelf. (These are tidally-corrected, which precludes their use in the DROT method.).

We used Tikhonov regularisation for each of the terms ${{{\mathcal{J}}}}$ and ${{{{\mathcal{J}}}}}^{{\prime} }$, though with distinct forms. For *ξ* ∈ (*ϕ*, *C*):

2 $${{{\mathcal{J}}}}(\xi )=\int_{\Omega }| {{{\boldsymbol{\nabla }}}}\xi {| }^{2}d\Omega$$

3 $${{{\rm{and}}}}\ {{{{\mathcal{J}}}}}^{{\prime} }({\xi }_{i},{\xi }_{i-1})=\int_{\Omega }| {\xi }_{i}-{\xi }_{i-1}{| }^{2}d\Omega .$$

The first provides a constraint on the smoothness of the solution fields while the second constrains the deviation of each from the value at the previous timestep. Regularisation parameters were selected based on L-curve analysis ^51^, with *α*_*C*_ reduced by a factor of 10 to allow for small-scale spatial variability in *C* such as might be produced by localised regrounding. We used a value for ${\alpha }_{\phi }^{{\prime} }$ from the literature^52^, but set ${\alpha }_{C}^{{\prime} }$ to zero to allow for maximum temporal variability in *C*. We started with an initial guess for *C* such that, at 4000 m a^−1^, the basal stress (with a linear sliding law) would have been 10 kPa over the ice shelf.

We used a standard form of the rate factor *A*(*T*) ^53^, with an internal energy field generated using a 100000 year calculation in which surface temperature, thickness and velocity were held at present day values and the combined ice temperature and moisture fraction field *E* = *CT* + *Lw* evolved toward equilibrium. The geometry was based on BedMachine-v3^54^, with time-evolving calving front positions extracted from Sentinel-1 backscatter images. The calving masks were applied after each calving event, though frontal advance was not allowed.

### Diagnostic simulations

To quantify the sensitivity of ice shelf flow to localised basal traction, we performed a suite of diagnostic simulations. These were initialised in May 2018, assuming a fully floating shelf. This involved solving an inverse problem for *C* on the grounded part of Pine Island Glacier (without imposing grounding on any of the floating ice) and *ϕ* everywhere. This inverse problem is distinct from those discussed in Sec. 4.2 and was used purely for model initialisation purposes.

We artificially imposed grounding over a small region of the ice shelf by raising the bedrock beneath a region corresponding to a typical grounded area observed in 2018 (shown in Fig. 2a), and prescribed non-zero basal friction coefficients (*C*) over this patch. We then solved (1) to find the ice speed over Pine Island Glacier with this additional sticky patch on the ice shelf. The bedrock over this region had to be raised by an average of 150 m. This suggests a significant underestimation of bedrock elevation in the region, as can be seen in Fig. 1b (white star), which shows grounding to have most frequently occurred on what the bedrock data suggest is a local bathymetric low point.

We used a regularised-Coulomb sliding law of the form:

4 $${{{{\boldsymbol{\tau }}}}}_{b}=-C\times {\left(\frac{| {{{{\bf{u}}}}}_{{{{\rm{b}}}}}| }{| {{{{\bf{u}}}}}_{{{{\rm{b}}}}}| /{u}_{r}+1}\right)}^{m}\frac{{{{{\bf{u}}}}}_{{{{\rm{b}}}}}}{| {{{{\bf{u}}}}}_{{{{\rm{b}}}}}| }$$

with a Weertman-like exponent of *m* = 1/3 and a threshold ice speed of *u*_*r*_ = 300m a^−1^ that represents a transition between viscous and plastic sliding^36^. We chose values of *C* so that, at the observed speed a which the glacier was flowing over the imposed grounded region, eq. (4) gave stresses of: 0, 1, 5, 10, 20, 30, …, 150, 175, 200, 250, 300, 400, 500, 750 and 1000 kPa. Of course, the choice of *C* changes the speed that the model predicts. Therefore, the stresses that the ice experiences differ compared to the target stresses. For example, Fig. 3 shows that our choice for *C* aimed at producing stresses of 150 kPa actually produced 148 kPa and 1 MPa produced 496 kPa, etc.

The resulting changes in surface velocity were compared to observed interannual variability to assess whether such stresses would have been detectable in the timeseries data.

### Advecting grounded spots

To track areas of the ice shelf that were observed to have regrounded, we remapped the masks delineated using the DROT method with the ice flow using satellite-derived velocity data. To do this, we required heavily smoothed velocity fields. We used a smoothed version of the 1996–2016 velocities given by MEaSUREs^55^ for the period prior to the Sentinel-1-derived observations starting from 2015^56,57^. Thereafter, we updated the velocity at each timestep according to a linear fit through a time series of ice velocity given by ENVEO^34^ (with mean taken over a region of the central shelf). To keep track of the regions beyond where velocity data were available (approximately at the calving front), we extrapolated ice velocities along the approximate direction of flow.

Remapping was performed using OpenCV’s remap function between each timestep^58^. This makes the assumption that velocities are constant along trajectories between timesteps. This is justified by the fact that timesteps are on the order of months to years and the very low strain rates observed on the central shelf. Remapping does some interpolation as transformations are not in integer numbers of pixels, so binary images tend to blur after many timesteps. To mitigate this, we thresholded the images after each timestep to create new binary images.

### Estimating tensile stresses due to elastic bending

**Fig. 8:**
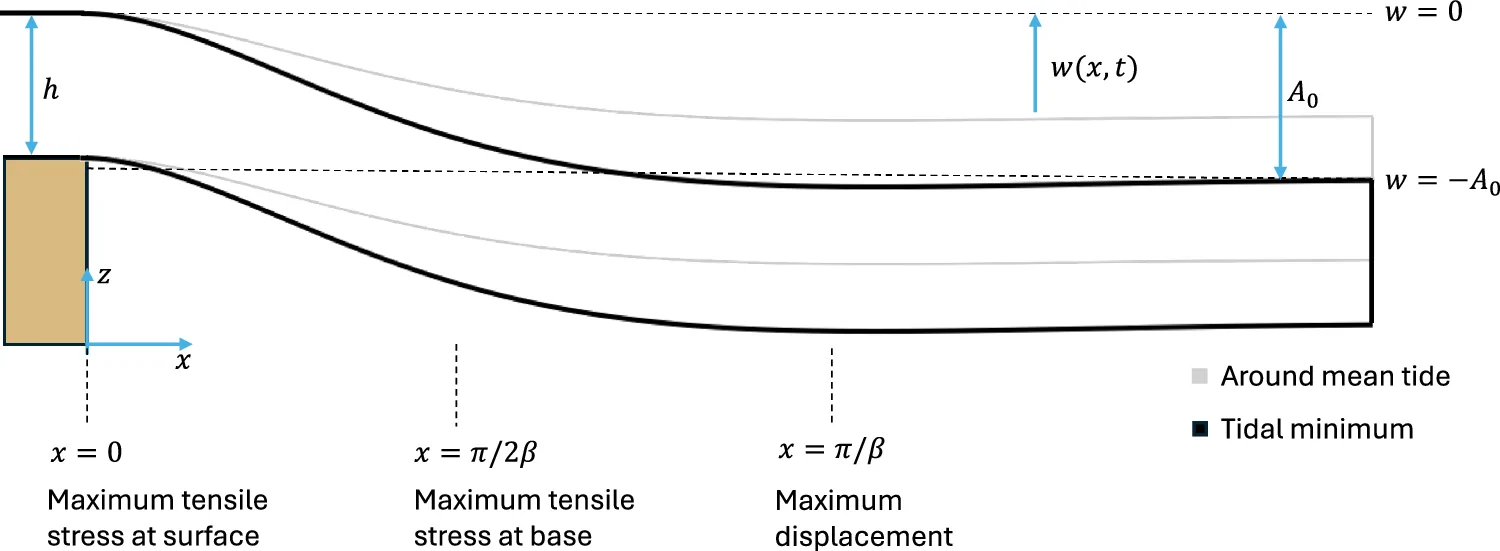
Schematic representing the flexure of a one-dimensional elastic ice shelf of thickness *h* with the motion of the tide. The tidal range is *A*_0_, the displacement of the surface from high-tide is *w*(*x*, *t*), the wavenumber characterising the solution for *w*(*x*, *t*) is *β*. Three locations in *x* are noted: *x* = 0 - the hinge line where stress is maximally compressive at the base and tensile at the surface; $x=\frac{\pi }{2\beta }$, where stress is maximally compressive at the surface and tensile at the base; and $x=\frac{\pi }{2\beta }$, where the displacement of the surface is maximum.

The bending of ice due to tidal motion has long been treated as a problem in elastic statics, with known solutions^59^ first applied to floating ice shelves in the 1950s and 1960s^40,60^. This beam theory has been subsequently widely studied with applications to real ice shelves augmented by a variety of observational methods for extracting data on ice shelf flexure^41,61–67^. Here, we use such a model to investigate whether tidal bending could generate stresses of sufficient magnitude to promote fracture or drive the growth of existing cracks on Pine Island Ice Shelf. We adopt a one-dimensional elastic beam model^40^, and consider the case in which ice remains grounded for a full tidal cycle, as we interpret for the locations identified by DROT. We take the origin *x* = 0 to mark the edge of a grounded region (Fig. 8).

Well-known solutions in mechanics allow us to write the longitudinal stress at height *z* above the neutral axis (line of zero stress) as:

5 $${\sigma }_{x}x(z,x,t)=\frac{Ez}{1-{\nu }^{2}}\frac{{\partial }^{2}w(x,t)}{\partial {x}^{2}}$$

where *E* is the Young’s modulus of ice, *ν* is the Poisson’s ratio and *w* measures the downward deflection of the ice from its position at high tide.

The deflection of the ice shelf can be written^40^:

6 $$w(x,t)={A}_{0}(t)\left(1-{e}^{-\beta x}(\cos \beta x+\sin \beta x)\right)$$

where the characteristic wavelength of decay and oscillation depends on thickness and material properties of the ice:

7 $$\beta={\left(\frac{3{\rho }_{w}g(1-{\nu }^{2})}{E{h}^{3}}\right)}^{\frac{1}{4}}$$

where *ρ*_*w*_ = 1027 kg m^−3^ is the density of seawater, *g* = 9.81m s^−2^ is the acceleration due to gravity, and *h* is the thickness of the ice.

Hence, it can be seen that

8 $${\sigma }_{x}x(z,x,t)=\frac{2{A}_{0}(t)E{\beta }^{2}z}{1-{\nu }^{2}}{e}^{-\beta x}(\sin \beta x-\cos \beta x).$$

This clearly has many extremal points, with the two of maximum amplitude at the hinge line (*x* = 0), where longitudinal stresses are extensional at the surface and compressive at the base, and at $x=\frac{\pi }{2\beta }$ where stresses are compressive at the surface and extensional at the base. The magnitude of stress at the latter is smaller by a factor of *e*^−*π*/2^ than at the former.

To calculate potential stresses induced by tidal bending, we took the ice shelf to be of uniform thickness of *h* = 550 m (the average thickness in the yellow area shown in Fig. 3a), *ν* = 0.3 and a value of *E* = 1 GPa. There is some variety of values for the Young’s modulus discussed in the literature, from 0.5−3 GPa ^41,64,68^. However, it is argued that these are often over-estimated from observational data given viscous effects are rarely accounted for^67^. Hence, we opted for a round value at the lower end of the spectrum. The lower stress compensates for the fact that the elastic model neglects viscoelastic relaxation, which would otherwise reduce stresses over tidal timescales, and ensures that our estimates remain relatively conservative. These choices result in a wavenumber of *β* = 6.38 × 10^−4^ m^−1^. The CATS2008 tide model^42^ shows that daily tidal ranges (maximum tide height minus minimum) in the centre of Pine Island Ice Shelf can vary from a few centimetres to 2 metres, however are concentrated close to the mean of approximately 1 m (FIg. 5b). For a point on the centre of the ice shelf, the daily tidal range between 2014 and 2024 according to the model was 1.04 ± 0.38 m. The calculations below are carried out for this range of tide amplitudes.

With the parameter choices defined above and tidal ranges predicted by the tide model, the maximum tensile stress at the surface is 209.3 ± 78.9 kPa, depending on tidal amplitude, and occurs at *x* = 0. The maximum tensile stress at the base of the ice occurs at a distance of 2.46 km from the hinge line and ranges between 43.5 ± 16.4 kPa.

## Supplementary information


Peer Review file


## Data Availability

All necessary data and configuration files to run the modelling experiments presented here, as well as the grounded regions we identified in shapefile format, the evolution of rifts R1-4 in shapefile format, the Landsat image analysed in Fig. 4, and all range-displacement anomaly maps used in the detection of the grounded regions have been deposited in the Zenodo database: https://doi.org/10.5281/zenodo.20748905.

## Code availability

The BISICLES Ice Sheet Model is open source and the code is available at: https://github.com/CPOM-Land-Ice-Modelling/bisicles-uob.
