# Supplementary material for: The dynamic response of Pine Island Glacier to two decades of intermittent ice shelf regrounding
Source: Nat Commun. 2026 Jul 29;17:9183. doi: 10.1038/s41467-026-75648-8 (PMC13522483; doi:10.1038/s41467-026-75648-8)
Supplement: Supplementary file 1 [file 41467_2026_75648_MOESM1_ESM.pdf]

nature portfolio

Peer Review File

The dynamic response of Pine Island Glacier to two decades of intermittent ice shelf regrounding

Corresponding Author: Dr Trystan Surawy-Stepney

This file contains all reviewer reports in order by version, followed by all author rebuttals in order by version.

Attachments originally included by the reviewers as part of their assessment can be found at the end of this file.

Version 0:

Reviewer comments:

Reviewer #1

(Remarks to the Author)
Impact of two decades of intermittent regrounding on the dynamics of Pine Island Glacier, Antarctica
Trystan Surawy-Stepney et al.

This manuscript investigates the regrounding of the Pine Island Glacier (PIG) ice shelf on bathymetric highs and its influence on the mechanical stability of the ice shelf. My interpretation of the argument is as follows:
- Grounding on bathymetric highs occurs due to heterogeneities in the bed at the grounding line and in basal melt rates, producing thickness variations that propagate downstream.
- However, neither historic surface velocity data nor ice sheet modelling provide evidence that such regrounding increases buttressing; basal shear stresses in the newly grounded areas do not appear to increase.
- Instead, contact with bathymetric highs appears to promote increased rifting, driven by ice shelf hinging in response to tidal motion, which in turn leads to elevated calving rates.
- Previous numerical modelling study suggests that this calving contributed to dynamic ice loss from PIG between 2017 and 2020.

Overall, the study is a valuable contribution, particularly in highlighting the perhaps counterintuitive result that regrounding does not necessarily enhance mechanical "stability" and may even have destabilising effects through tidal hinging, and I would recommend publication in Nature Communcations. The manuscript is generally clear, well-written, and the analyses appropriate for addressing the central aim.

My main observation is that the manuscript contains two key conclusions: (1) regrounding does not necessarily reinforce the mechanical stability of the ice shelf; and (2) regrounding can potentially lead to mechanical weakening. The first point is well-supported; the second (on the role of tidal hinging in driving calving) is presented as a key mechanism but is more speculative. The robustness of this mechanism is not fully established, leaving me uncertain about its role relative to other processes (particularly given the final paragraph in section 4.1). This is definitely an important mechanism to highlight – and if tidal hinging is truly responsible for the development of rifts and ultimate calving of PIG (which seems reasonable to conclude), then the modelling community needs to put more effort into capturing the dynamics correctly.

However, I would recommend tightening the manuscript's focus on the first conclusion: that regrounding does not necessarily strengthen the ice shelf mechanically. Where the mechanism of tidal hinging is discussed (particularly in Sections 4.1 and 5), it should be clearly framed as a potential explanation for observed calving rather than a conclusive driver. And I'd strongly recommend that section 5, in particular, be revised to reduce vague and speculative language (e.g., "intuitive," "natural to assume," "suggests… this is optimistic", "We leave these details to a future in which we can simulate the full viscoelastic behaviour of the ice shelf", "accurate prediction of future mass loss from the Antarctic Ice Sheet demands more subtle numerical modelling than previously thought"), which currently dilute the manuscript's main message. Clarifying these points will help convey the main finding from this study: that regrounding does not necessarily mechanically strengthen ice shelves.

I otherwise had only a few specific comments:

- The final two paragraphs of the introduction could be a bit tighter with wording to improve clarity. E.g. at L46 "...reflecting changes to the bed topography at the GL as it migrates" – I originally interpreted this to mean landscape evolution, but do you mean spatial heterogeneity in the bed topography and how it influences the ice thickness? At L46-55, I recommend to precisely define "unsteady" and "stable/unstable" where used (i.e. for unsteady do you mean that it's changing rapidly? For stability/stabilising do you mean mathematical stability of the grounding line or the propensity for large-scale, rapid retreat).
- Introduction: you could also cite Reed et al. (2024) who use a numerical ice sheet model to investigate evidence of marine ice sheet instability during past retreat events of PIG
- L173: "...due to tidal motion in the vicinity of the grounded locations". Is it possible to be more precise here – "downstream of the grounded locations"? Also could potentially link this to where we expect passive vs active ice regions of the ice shelf?
- Section 6.2. How sensitive are the results to the regularisation parameters? Is 500 m sufficiently fine resolution to capture variations in the basal shear stress? I also wondered about the SSA equations and whether some of the other components of the stress tensor not resolved might be relatively more important where you reground.

Reference

Reed et al. (2024), doi: 10.1038/s41558-023-01887-y

Reviewer #2

(Remarks to the Author)

Title: "Impact of two decades of intermittent regrounding on the dynamics of Pine Island Glacier, Antarctica"

Authors: Trystan Surawy-Stepney, Benjamin J. Wallis, Anna E. Hogg, Ian J. Joughin, Stephen L. Cornford, Pierre Dutrieux

GENERAL COMMENTS:

This paper is about the numerical modeling results of Pine Island Glacier's regionalized basal drag at a bathymetric high in the ice shelf and whether the grounding that has taken place in the past over this bathymetric high has had any impact on the ice shelf's dynamics. The paper does not feel fully developed and is difficult to follow at times. I highlight both of these concerns in the following paragraphs and in the line comments. At this time, I do not recommend that this paper be accepted for publication.

It is incredibly important that the authors stress the differences between the ice sheet's grounding line and the ice shelf's ice rise grounding line. The use of the word "regrounded" is often ambiguous to the various spatial grounding lines referred to throughout the abstract and the entire manuscript. The authors need to very obviously emphasize that the "regrounding" is in reference to the ice shelf's ice rise and at no point in the manuscript do the authors touch on any changes to the ice sheet's grounding line. In the detailed comments before, I have highlighted every instance where it is unclear what part of the ice shelf is being "regrounded". Additionally, the authors need to provide a definition of "regrounding" and "grounding" relative to the manuscript because it is not clear if either term is a fixed state, temporary, or reoccurring. This is especially true with the comment made on Line 70 about "ephemeral" grounding.

The data being presented in the manuscript's current state makes the implied conclusion that the ice shelf's ice rise role in PIG's grounded ice dynamics an extreme reach.

I understand the thought process behind why basal drag was modeled, but there are several other means of assessing the bathymetric high's effects on the ice shelf that rely primarily on actual data, so I wonder if the methods implemented in this project are overly complicated and considering the amount of smoothing that seemed to have been applied to the data, maybe alternative assessments would be more impactful and accurate in the final analysis. It seems that despite there being observational data available from a multitude of sources over the timeline of the study, that what the authors have done is model basal drag from modeled or "idealized" coefficients and then used that value of basal drag to model the surface velocity for valuating the impact of the bathymetric high. Considering there is multitemporal velocity, elevation (e.g., REMA, ASTER, OIB), and some ice thickness—which can be used for validating hydrostatic thicknesses from surface elevations—datasets, why wasn't a more direct means of estimating basal drag like van der Veen and Whillans (1989) or MacAyeal (1993) used? Lines 107-112 leave the reader doubting how realistic the modeled results are. More importantly, why was only basal drag presented? The stress with the greatest impact on rift propagation is lateral shear. Why wasn't shear stress, or at bear minimum, shear strain, calculated/presented in the results? What about surface elevation changes of the ice shelf? There should be plenty of REMA data over the timeline of this study and you already have surface velocities, so calculating Lagrangian vertical changes in ice thickness should be relatively straight forward. The heigh above buoyancy over the bathymetric should really should be calculated at each grounding time step (or with a temporal reference that is as close to the InSAR data as possible) as another means of supporting the argument that grounding is taking place.

I am confused about what exactly is being used for parameters in the BISICLES (Cornford et al., 2013) algorithm. In solving for basal drag, surface elevation, surface slope, and ice thickness are required inputs. Based on the provided text, it seems that those parameters only come from BedMachine v3 (Morlighem et al., 2022)—is this the case? If so, then the authors need to provide something of support either in the form of additional model runs or from the literature to show that using a temporally static dataset still produces accurate basal drag results. According to Bevan et al, (2023), using a constant BedMachine ice thickness does result in reduced ice velocities in temporally dynamic model runs. As that study represents a timeline of modeled results significantly longer than this one, I do not know what the full impact using a temporally static dataset will have on the accuracy of this study's results.

I would like to know why the ice sheet's grounding line was not estimated along with the bathymetric high's grounding line. Surely the location of that major boundary is playing a roll in the grounding of down-flow, isolated grounding. I may be wrong, from the Rignot et al, (2016) grounding lines, it looks like the 2011 grounding line is in use for the numerical modeling. What evidence do the authors have to support using a temporally static boundary for the basal drag estimates?

In Sections 2-4, both results and discussion points are included in each section. It would be better to parse results from discussion points because the discussion points are written very passively and it takes away from impact of the results.

LINE COMMENTS:
Line 4: It is unclear here what exactly is being "regrounded" if the authors are referring to the ice sheet grounding line or the ice shelf ice rise grounding line.

Line 5: Refer to a general "hypothesis" in the previous sentence before using "this hypothesis" in this one.

Line 8: "that this had on the dynamics of the glacier"—this is a misleading statement. The only results that illustrate any form of generated flow assessment is Figure 3a and that barely shows any of the grounded ice. It also seems like there is a much better way of highlighting the secondary influences rather than referring to them as "indirect".

Lines 9-10: "regrounding"—regrounding of what? "it likely contributed to the promotion"—there was virtually no evidence at all to support this statement. Saying something less definitive like, "one potential driver behind the 2015-2020 calving was the possible…" is more reflective of the presented results. "held largely responsible"—what was? It's probably in reference to "regrounding" but it's not obviously that that is the case.

Line 11: "regrounding"—of what? "systems"—way too broad of a term. Be more precise and use phrasing specific to glaciology/marine terminating glaciers/ice shelves/etc.

Line 14: Making a bold statement that PIG is "currently" losing the most mass of any glacier in the world needs to have a much more up to date reference than the 2017 data used in Rignot et al, (2019). Additionally, double check that in the last few years Thwaites hasn't bypassed PIG for that title of most mass loss.

Lines 20-22: This sentence needs a reference, or say something along the lines that the authors have observed said changes. "The early stages of the retreat are difficult to characterise"—why? If due to a lack of observations, then say that. Also, include an approximate range of time when the retreat began.

Lines 23-25: "perhaps aided by a lack of available intermediate steady-state grounding line positions"—what does this mean?

Line 24: Delete the 's' from "shows".

Lines 26-28: During those dates provided, Joughin et al, (2016) reports that this is when the island formed and not the peninsula.

Line 30: "this ice keel"—up to this point, a specific ice keel has not been referred to, so "this ice keel" is confusing.

Lines 31-45: The background information is useful, but it is misleading for the reader because all mentions of the grounding line are with respect to the ice sheet grounding line and has nothing to do with the ice shelf ice rise grounding line. Considering the direction of the paper, this paragraph should only be referencing facts about the ice shelf and nothing to do with the ice stream or the ice sheet grounding line.

Lines 46-55: Like the previous paragraph, the references to the ice sheet's grounding line are confusing because this study literally does not include a single bespoke observation of PIG's grounding line. Ice sheet grounding line inferences need to be removed. Details about past observations of changes to PIG's pinning points or shear stresses are actually relevant to this study.

Lines 46-48: "system"—use a different word that is specific to ocean-terminating glaciology. "changes to the bed topography"—the bed topography is changing? Unless sediment deposits hundreds of meters thick are taking place, this seems unlikely. "different parts of the ice"—use more unambiguous language.

Lines 49-51: Make sure to include that the Bindschadler reference is with respect to PIG and not glaciology as a whole. "the imprint on the fine structure of the thickness"—what is this? If this is implying that past flow conditions of grounded ice can be observed from the surface of an ice shelf, then Hulbe and Fahnestock (2007) is a better source to cite. "grounding line geometry"—geometry of what? Glacial ice?

Lines 51-52: "natural to assume that positive perturbation in the thickness"—this is confusing. Are there perturbations horizontally homologous? Is the areal coverage over the entire glacier, grounded, or floating ice? Short-lived increases in ice thickness at an ice sheet's grounding line could actually be evidence of mass imbalances of ground ice flow increases causing greater amounts of grounding line ice discharge like what has been observed on the Crosson Ice Shelf (Mouginot et al., 2014).

Line 60: These displacements vertical or horizontal?

Line 62: “would”—use more active language.

Lines 68-69: The citations are confusing. Did Kimura et al, (2016) produce the data in the state with which it was used in this study? If not, then there’s no point in citing that body of work here.

Line 70: “hot spot”—use a different phrase. Up to this point, there has been no evidence provided that shows the bathymetric high is a point of ice damage, so “hot spot” is confusing. “ephemeral”—so, how do the authors actually define what grounding is? How does this differ to “light grounding” referred to on Line 82.

Lines 71-73: “flow velocity”—where does this data come from? “to keep track of which parts of the ice shelf have been grounded at different times…”—this is ambiguous language and too simple for a journal article. Concisely tell the reader that following the ice shelf grounding experienced by those spatially discrete regions, remapping techniques were used to temporally track the ice columns to their present-day locations.

Line 73: In Figure 1’s current state, it is untrue that regionally discrete, temporal ice shelf grounding is being showcased. See specific comments about the figure.

Line 75: With the reference to “thinning” here, were surface elevation changes assessed in this study? There are much more current studies (e.g., Davidson et al, 2023) using data newer than 10 years old as stronger evidence to support whether thinning has taken place.

Line 76: “This is also evidenced…”—what is also evidenced? 4-year consistent floating?

Line 85: “The methods we use”—too broad and passive. Say, “Our InSAR processing requires…”

Line 86: “We emphasize the difference…” Where? How? Did I miss this somewhere?

Line 89: “…observation that grounding appears to have occurred…”—passive language. Say, “…interpretation that grounding occurred…”

Lines 90-91: “…perhaps corresponding to periods of large tidal amplitude, or diminished mean sea levels due to regular oscillations in atmospheric pressure.”—Do either of these parameters actually statistically correspond to the timeline of grounding? If not, this is both extremely passive language and irrelevant to the observations.

Line 94: “grounding”—grounding of what? “influence”—influence of what?

Lines 99-101: I am confused by this. Why is it necessary to “raise the bedrock”? If Schoof and Hindmarsh (2010)’s shallow shelf approximation is what is being implemented in the BISICLES, then this shouldn’t be necessary. It seems too that if the bedrock was “raised” to touch the bed of the ice shelf, then the surface relief will also need to be altered to mirror the bed geometry the ice is flowing over in which case driving stress and ultimately basal drag will also be affected. Cornford et al, (2015) does mention that some previous studies have modified the bedrock elevations so that ice thicknesses match discharge rates at the ice sheet grounding line, but this is not an equivalent scenario for the ice shelf bathymetric high. Additionally, it is not clear what the spatial location of where raising the bedrock took place.

Line 102: “namely” is an awkward word choice.

Lines 102-103: How do the authors know that using nearly static terminus positions produced accurate results?

Line 127: “grounding during this period”—what exactly this is timeline?

Lines 130-132: This reads as though the BISICLES and thus the Shallow Shelf Approximation was the wrong choice of model for this study…

Line 134: This section title reads as though an analysis is carried out, but that is not the case and no real conclusions about the “role of regrounding” are determined. This section needs to be renamed to reflect the purely observational nature of it.

Line 135: Lines 126-127 directly contradict this. Additionally, “glacier dynamics” is not the same thing as ice shelf dynamics and should be altered. The authors have not modeled any of the grounded portion of PIG’s stress regime to be able to make a comment on whether the glacier’s dynamics have been altered.

Lines 136-138: PIG is sensitive to changes in its lateral stress. To say that it is sensitive to changes in the terminus is misleading.

Line 139: “pattern of calving” is ambiguous. Refer either to the calving rate or the areal region of calving events.

Line 140-141: Replace “margin” with “terminus”.

Line 148: “this unfamiliar mechanism”—referring to the 2018 calving event, or the central origin of rifts? “usually associated with rift initiation at the ice margins”—the way this is written, it needs a reference. If it is instead more specific to PIG calving, then include something about how this is an observation of the authors.

Line 152: “We investigated the spatiotemporal coincidence of rifting”—in what sense? This part of the sentence made me think that geostatistical analyses were going to be conducted. It is more appropriate to simply say that observations were made. “with the grounding of ice”—what “ice” is being referred to here?

Line 158: Just because the rifts are first observable down-flow of the bathymetric high it does not necessarily mean that they are the result of a grounding event. Use "down-flow” instead of “wake” here and on Line 159. Particle tracking has been used to assess horizontal displacements of the grounded regions—it should be reasonable to assign a grounded event to a rift if the assumption is made that the rift’s initial fracture first propagated during the grounding event.

Line 164: How do you know that all surface depressions correspond to rifts or basal crevasses? I agree that what is being identified in Figure 4c is showcasing rifts, but not basal crevasses. The OIB depth radar shows clearly what are crevasses and what are rifts. It is necessary to support this interpretation with either literature or data that the surface depression are one or the other or both.

Lines 171-172: Radar data shows that a rift can exist without an overlaying surface expression. This can be observed from the CReSIS data flown over Petermann Glacier. Additionally, a surface expression will not appear until an ice shelf reaches a specific thinness. What that exact ratio is is unknown, but studies like Humbert et al, (2015) and Child et al, (2021) have shown that large bottom fractures can exist for significant periods of time before a surface expression appears.

Lines 172-173: The main flaw in this hypothesis is that the 2015-2020 rifts did not occur with exact timing or at equal intervals. This indicates that there were deviations in the stresses acting on the ice shelf which would not be the case with simply tidal hinging as we would expect that to behave with consistent force.

Line 178-181: Why is an ice thickness of 500 m being assumed? Why is it not being estimated from surface elevations or airborne radar? Are any of these parameters specific directly to PIG? If not, then the 188 kPa simply shows that the stress regime COULD be such that fracturing might occur. This value is not temporally relevant and therefore could be reflective of any non-grounded deviatoric stresses.

Line 182: “elsewhere in the literature”—provide examples.

Lines 183-184: The Vaughan et al, (2012) comparison is misleading. The Vaughan et al, (2012) details a completely different fracturing scenario for fracturing that is occurring parallel to ice flow and is the result of hydrostatic adjustments causing very shallow (both surface and bottom) fracture propagations. There is no evidence provided here or in the literature that supports that tidally-induced hinging stresses will be nearly the same as those generated from hydrostatic readjustments. None of the fractures reported in Vaughan et al, (2012) had penetrated the entirety of the ice shelf’s ice thickness. I would argue that the deviatoric stresses reported in Vaughan et al, (2012) are absolutely not indicative of the intermittent burst of stress deviation required for a mid-ice-shelf-full-fracture to take place.

Line 187: What is the crack tip in this study’s scenario? Is the implication that the rift formed from a basal crevasse? If so, that needs to be said.

Line 189: Shouldn’t the stresses be greater than the fracture toughness?

Line 191: This is an inappropriate reference as it pertains to freshwater ice. The most thorough studies of glacier ice fracture toughness are the Rist et al, (1999) and Rist et al, (2002) studies.

Lines 196-197: I would argue that it is possible to have a more thorough assessment here as to why no rifts have appeared in post-2017 grounded ice shelf ice. The authors have an impressive amount of velocity data and there therefore have the ability to produce a comprehensively extensive record of the changes in strain rates and tensile stresses for the entire ice shelf. There were likely short-lived events that affected the stress regime and those events will be captured in the velocity record. Additionally, the particle tracking should be extended to when the bathymetric high grounded ice was initially flowing over PIG’s ice sheet grounding line.

Lines 203-207: Both of these examples are irrelevant to this study. At no point do the authors in this study refer to the ice sheet grounding line. I fully believe that it is important to include it in a study like this, but in the current state of the manuscript, any comparisons to the ice sheet grounding line is unrelated to the present analysis. As stated earlier, the Vaughan et al, (2012) basal crevasses formed laterally—it is a completely separate stress regime than what is observed in this study.

Line 275: Write out the BISICLES acronym.

Line 277: Over what extent was the optimization problem solved for?

Line 296: Monthly observations of what? How do these velocities compare with those from Line 252?

Line 322: Include what years the pre-2015 velocity data is from.

Line 324: Why was it necessary to keep track of regions of ice that had calved?

FIGURES:
The attached pdf has my comments with regards to the paper's figures.

REFERENCES
Child, Sarah F., Leigh A. Stearns, C. J. van der Veen, and Pedro Elosegui. "Basal crevasse formation on Byrd glacier, East Antarctica, as proxy for past subglacial flooding events." Geophysical Research Letters 48, no. 15 (2021): e2020GL090978.

Cornford, Stephen L., Daniel F. Martin, Daniel T. Graves, Douglas F. Ranken, Anne M. Le Brocq, Rupert M. Gladstone, Antony J. Payne, Esmond G. Ng, and William H. Lipscomb. "Adaptive mesh, finite volume modeling of marine ice sheets." Journal of Computational Physics 232, no. 1 (2013): 529-549.

Cornford, Stephen Leslie, D. F. Martin, A. J. Payne, E. G. Ng, A. M. Le Brocq, Rupert M. Gladstone, Thomas L. Edwards et al. "Century-scale simulations of the response of the West Antarctic Ice Sheet to a warming climate." The Cryosphere 9, no. 4 (2015): 1579-1600.

Davison, Benjamin J., Anna E. Hogg, Noel Gourmelen, Livia Jakob, Jan Wuite, Thomas Nagler, Chad A. Greene, Julia Andreasen, and Marcus E. Engdahl. "Annual mass budget of Antarctic ice shelves from 1997 to 2021." Science Advances 9, no. 41 (2023): eadi0186.

Humbert, Angelika, Daniel Steinhage, Veit Helm, Sebastian Hoerz, Jacqueline Berendt, Elke Leipprand, Julia Christmann, Carolin Plate, and Ralf Müller. "On the link between surface and basal structures of the Jelbart Ice Shelf, Antarctica." Journal of Glaciology 61, no. 229 (2015): 975-986.

Joughin, Ian, David E. Shean, Ben E. Smith, and P. Dutrieux. "Grounding line variability and subglacial lake drainage on Pine Island Glacier, Antarctica." Geophysical Research Letters 43, no. 17 (2016): 9093-9102.

MacAyeal, Douglas R. "A tutorial on the use of control methods in ice-sheet modeling." Journal of Glaciology 39, no. 131 (1993): 91-98.

Mouginot, J., E. Rignot, and B. Scheuchl. "Sustained increase in ice discharge from the Amundsen Sea Embayment, West Antarctica, from 1973 to 2013." Geophysical Research Letters 41, no. 5 (2014): 1576-1584.

Rist, M. A., P. R. Sammonds, S. A. F. Murrell, P. G. Meredith, C. S. M. Doake, Hans Oerter, and K. Matsuki. "Experimental and theoretical fracture mechanics applied to Antarctic ice fracture and surface crevassing." Journal of Geophysical Research: Solid Earth 104, no. B2 (1999): 2973-2987.

Rist, M. A., P. R. Sammonds, Hans Oerter, and C. S. M. Doake. "Fracture of Antarctic shelf ice." Journal of Geophysical Research: Solid Earth 107, no. B1 (2002): ECV-2.

Schoof, Christian, and Richard CA Hindmarsh. "Thin-film flows with wall slip: an asymptotic analysis of higher order glacier flow models." Quarterly journal of mechanics and applied mathematics 63, no. 1 (2010): 73-114.

Van Der Veen, Cornelis J., and I. M. Whillans. "Force budget: I. Theory and numerical methods." Journal of Glaciology 35, no. 119 (1989): 53-60.

Vaughan, David G., Hugh FJ Corr, Robert A. Bindschadler, Pierre Dutrieux, G. Hilmar Gudmundsson, Adrian Jenkins, Thomas Newman, Patricia Vornberger, and Duncan J. Wingham. "Subglacial melt channels and fracture in the floating part of Pine Island Glacier, Antarctica." Journal of Geophysical Research: Earth Surface 117, no. F3 (2012).

Reviewer #3

(Remarks to the Author)
Surawy-Stephney et al., present a holistic study looking at the effects on regrounding at Pine Island Glacier. This work is a potentially important contribution as the influence of intermittent regrounding of an ice shelf is a fairly novel topic but there are some concerns that need to be addressed. Whilst I think the article provides an interesting study, I do not think it is best suited to Nature Communications.
Overall, the writing style of the article could be improved. There are often clunky and difficult to read sentences and numerous references to demonstrative pronouns where it is unclear what the object is. I have pointed out some but there are lots of sentences that could be streamlined by removing unwanted parenthesis. I only point this out as some of the English detracts from the scientific messages.
I have several major concerns that I outline below:
1. Methods. It is unclear if imposed grounding is only on the diagnostic simulations or the inversions too. Reading the methods it seems clear that the imposed grounding ice is only for diagnostic simulations but in Section 3 of the main text it is unclear. I suspect this is due to a lack of distinction between 'imposed grounding' used in the diagnostic simulations and

‘natural grounding’ which occurred during the observed time of the inversions. It would help greatly if the authors could add some clarity to the text and add a couple of sentences defining each.
Diagnostic simulations – I am not opposed to the methods of moving the bedrock even on a real domain but it would it be good if the authors could explain the reasoning behind this i.e. explain why you don’t just thicken the ice shelf over the grounded and near grounded area.
It took me a couple of read throughs to understand the modelling methodology. As I understand –
1) Invert for C (ungrounded ice has no basal traction). Used for inversion results.
2) Raise bedrock over imposed area.
3) Prescribe values of C on the imposed grounded ice related to the basal stress from the Joughin et al., (2019) regularised Coulomb function.
Assuming the above is correct, the diagnostic simulations sections should be improved for clarity. The main text also confuses the order of the methodology and separation of the inverse and diagnostic simulations.
2. The role of regrounding in calving between 2015-2020. It is unclear what this section adds to the main manuscript. It is mainly a description of observations of rift formation of the area of regrounding. The descriptions seem reasonable, but it is vague it how these rifts produce calving. I suggest either the subsection title is changed to something like, ‘The role of regrounding in rift formation’ or more detail is added as to how/if these rifts produce calving. For example, what is the time for advection from the area of grounding to shelf wide rifting? Can the calving waiting times be linked to the timing of the regrounding?
Furthermore, a simple analysis of the 2D planar stress/strain field (presumably available from the BISICLES simulations with imposed grounding) could provide insights into rift propagation. Similar to the authors comments about the basal stress the magnitude is of secondary interest and instead the ice shelf stress pattern is important. Looking at Fig. 3a panel 4 I would be surprised if the stress contours did not replicate the observed rifting seen in Section 4. Such analysis could add weight to the study in terms of ice sheet calving. All calving functions, whether damage or stress- based, rely on a SSA-style stress field. If the viscous dynamics do not pick up any changes in the stress field then the authors arguments on tidal hinging become more potent.
3. Although I agree with the authors that tidal hinging is a potential source of failure, it is hard to justify the extent to which they have taken the argument without convincing modelling study. I would suggest this is instead phrased as a potential implication of the inversion/diagnostic simulations or a more detailed modelling study is done.
4. I think this is a really interesting study that the Nature format does not do justice to, particularly with regards to the methods. Additionally, a longer format article would allow the authors to expand their study in relation to calving which at the minute is purely speculative.
5. The nice modelling and observation results of the paper indicate regrounding does significantly impact ice velocity. This is an interesting result but it is not presented as the main conclusion. Instead the focus is on rift formation from ice shelf regrounding which comparatively less evidence is provided for.
Minor Comments.
Ln 31 what ‘things’?
Ln 44 ‘subverting’ not sure this is the best term.
Ln 46 ‘like this’ what is ‘this’ referring to?
Ln 49 Stylist. The sentence could be restructured to avoid back-to-back citations.
Ln 53 Rephrase. This is a difficult sentence to follow.
Ln 110 It is unclear if ‘fictitious’ stress refers to any basal stress on the ice shelf or only in the imposed regrounded area. Or is this purely referring to whether the inversion indicts regrounding based on the spatial pattern of the basal stress prior to imposing the regrounding?
Ln 135-137 This sentence is confusing and needs to be clarified. I think the full stop is also meant to be a comma
Ln 301 states the use of ‘time-evolving calving front positions’. Are these monthly constrained i.e. one front position for each monthly inversion or are you evolving the geometry whilst inverting? Ln 104 in the main text would suggest the terminus evolving.
Ln 306 how much do you have to raise the bedrock for each simulation? Is it within tidal range or does it rely or a much greater ice thickness shift? This would allow the reader to get a sense how likely regrounding is.
Ln 314 I would advise against using ‘fictitious grounding region’ perhaps imposed grounding region?
Ln 316 This sentence is awkward and difficult to understand.
It would also be useful for the authors to establish why they prescribed the friction on imposed grounded area rather than inverting.
Ln 322 Stylist. This could be rephrased to avoid the references backing into each other.
Figures. Larger text on axes.

Version 1:

Reviewer comments:

Reviewer #3

(Remarks to the Author)
I thank the authors for the changes to the manuscript that have improved it and agree that the revised manuscript is improved. After reading their responses and the new manuscripts, I have only some minor remaining concerns that need to be addressed.
Thanks for the additional explanation and changes to the text regarding tidal hinging promoting calving. I am supportive of the reframing of the conclusions that the primary conclusion (shown by the experiments in the manuscript) relate the lack of

buttressing provided by the regrounding. Although the tidal hinging in the paper presented is a possible explanation for the fracture initiation and growth, the major weakness of this study is its inability to directly link the ice sheet model with the predicted calving.
The redrafting of the inverse methods has substantially improved the manuscript. I now more fully appreciate the authors methods which I am supportive of. I have a couple of minor points I think would improve the manuscript.
• The assumption that the entire ice shelf is grounded in the first set of inverse experiments is stated in the main text, but it would be useful if it was very briefly noted in section 6.2 too.
• The distinction with the diagnostic simulations is much clearer now. Ln 325-326, "This inverse problem is distinct from those discussed in Sec. 6.2 …" should be altered as these results are presented in the main text rather than purely for initialisation. Perhaps instead state that theses experiments are conducted for a fixed timeframe.
I thank the authors for their explanation of how much they had to change the basal geometry to induce regrounding. It needs to be stated how uncertain the bathymetry is and by how much it was elevated. I don't see it as a weakness that it had to be adjusted so much if the data is so uncertain. This can probably be stated in the methods if the authors are uncomfortable with the impression this gives. However, justification for this (reasonable) method needs to be stated in the main text. The need for accurate bathymetry/bed geometry to model calving/basal stress is well known.
Overall, I think this paper makes a useful contribution to our understanding of the role buttressing plays in ice shelf dynamics and a potential source of fraction initiation. I would encourage the authors to further consider how such processes can be incorporated in icesheet models in the future.
Minor comments
Ln 107 '(they are all ficticious)'. This takes away from the fact that these experiments are reasonable and justifiable. Additionally, it would be useful to note here why they are notional values. I suggest something like, "which are notional/theoretical as grounding is imposed on the entire ice shelf. Instead, we focus…". Note fictitious is misspelt.
Ln 128-130. Should these not refer to Fig 3 and not to Fig. 2?
Ln 148-154. It is not clear what 'this' at the start of each sentence is referring to for all four out of the five sentences in the paragraph. Please rewrite for clarity.
Ln 196-198. Again with neighbouring sentences it is unclear what 'this' is referring to.
Ln 231. Change 'this' to 'regrounding' or' grounding'.

Reviewer #4

(Remarks to the Author)
This study deals with the analysis of the impact of intermittently grounding of the Pine Island ice shelf on high points in the subshelf bedrock over the last two decades. A noteworthy conclusion is that regrounding provided very little resistance to the flow of Pine Island Ice Shelf. Regrounding happened during a period of glacier speed-up and terminus retreat. Based on the results, regrounding may even have played a role in terminus retreat.

General comments:
1) Remote sensing part:
DROT vs DINSAR: A stronger case for the use of DROT over DINSAR would be warranted.
As written, the authors open themselves up to the question why they mention DINSAR, highlight its greater sensitivity, yet do not test the method. This particularly in light of the fact that by calculating offsets, 80% of the work for DINSAR is already done.

Crack monitoring and visibility: Clarify what imagery was used to monitor crack propagation and specify the resolution. Cracks may have formed and weakened the ice well before they are visible in imagery. Is there any impact of mixing SAR and optical SAR and optical data? Were the cracks manually outlined, what was your detection threshold? Does SAR image geometry play a role for visibility?

2) Modeling part:
The primary concern relates to the experiment in which the entire ice shelf is artificially grounded. It is unclear why the authors expect spatial variability in basal drag under these conditions. If the ice shelf is not in contact with the seamount, the velocity field across the shelf should remain relatively smooth, and substantial spatial variations in basal drag would seem unlikely.
In addition, while the authors report changes in basal drag (in kPa), they do not quantify the corresponding reduction in velocity. Although presenting basal drag alone is acceptable, the omission of velocity changes limits the interpretation of the results. Notably, they report a 5% reduction in speed when grounding only a small portion of the ice shelf. This raises the question of what impact fully grounding the ice shelf has in the first experiment, and why the associated velocity changes were not included.

Specific comments:

Line 23: 'the late 2000s', so does this mean that the retreat started in the 70s? This sentence is not very clear.

Line 25: 'available intermediate steady-state ...' What does it mean? What do you mean with steady-state? Isn't this because there are no observations during that period?

Line 31: 'during the 2000s'. Is this shown in Figure 1?

Line 56: what does 'positive' perturbations mean?

Line 67: "…combination of TerraSAR-X and Sentinel-1 data." Clarify that the data was processed separately

Line 75: 'in the 1980s'. Can you provide a reference for this claim?

Line 80: Why would thinning of the ice shelf and passage of a thick ice parcel over the bathymetric high point have the same impact, shrinking of the grounded area

Line 88: 'Thus one could imagine...' I don't understand this sentence. In the sentence before, you say that you are not 'confident' about past regrounding. Does this mean that ice would be progressively grounded over a deeper bed if we could go back in time and observe it?

Line 94: Sentinel-1 shortest repeat period. Recommend to mention 6 days.

Line 102: I am not sure I understand the need for saying 'each month'. Does this mean that the ice-shelf is grounded during the period 2016-2021?

Line 107: please define stickiness. Despite understanding what the authors refer to (coefficient C, also mentioned in the methodology), I don't think this is the correct terminology.

Line 110: What is the % impact on glacier velocity based on artificially grounding the ice shelf with 4kPa of basal drag? Later in this section, you quantify the slowdown due to partially grounding the ice shelf, but the same analysis for the first experiment is missing.

Line 111: 'For context ...' Do you have a reference for the 'representative stress scale'?

Line 113: Couple of hundred meters
Very casual formulation.

Line 115: 'While the recovered ...' I don't understand why you would expect some spatial variability in the inferred friction coefficient, given that the velocity map is relatively smooth over the ice shelf.

Line 195: this paragraph is confusing. Please see my comments below.

Line 196: 'This suggests ...' this sentence is somewhat confusing. What is the 'hydrological' connection between a weekly deformable till and 'low effective pressure'. I may have missed this, and I apologize if I did, but this should be made more clear for the reader.

Line 199: 'unwise'? I don't understand this.

Line 200: 'the merits of effective-pressure-dependent sliding laws'. I am not sure I understand. In section 3, you say that grounding the ice shelf (using which friction law?) does not change velocity over the grounded glacier. If that is the case, I am not sure you can claim the 'merits' of this type of friction laws from the results shown here. I do agree on the importance of including effective pressure, but this goes beyond the scope of this manuscript. Again, I may have misinterpreted this section, and I apologize if I did. I would appreciate some clarification.

Line 240: Zenodo link is missing.

Line 280: What friction law do you use to compute velocity changes? ""

Figures:

Figures 1,3,4: please consider using a colorblind-friendly colorbar that is different from jet.

Figs 3 & 4:
Suggest that every panel gets a letter.

Figure 4: it is hard to see the bathymetry contour.

Reviewer #5

(Remarks to the Author)
I co-reviewed this manuscript with one of the reviewers who provided the listed reports. This is part of the Nature Communications initiative to facilitate training in peer review and to provide appropriate recognition for Early Career Researchers who co-review manuscripts.

Version 2:

Reviewer comments:

Reviewer #3

(Remarks to the Author)
This is the third time I have reviewed this manuscript. I am happy the authors have incorporated my suggestions and agree about the limitations of the article (mainly that the modelling components are distinct from the observations). I think this is an interesting study that suggests a potential novel mechanism for the observed calving at Pine Island Glacier. However, the study cannot conclusively demonstrate that regrounding leads to calving given the lack of a transient ice sheet model in the study. Despite this, I am supportive of the conclusions which are now framed in a way that acknowledges the limitations of the modelling whilst providing a reasoned argument that tidal hinging could have led to fracture initiation at Pine Island Glacier.

Reviewer #4

(Remarks to the Author)
This manuscript was co-reviewed by myself and a Postdoctoral Researcher in our group. This is part of the Nature Communications initiative to facilitate training in peer review and to provide appropriate recognition for Early Career Researchers who co-review manuscripts.

Thank you for addressing our comments and making an effort to provide detailed responses to each issue in addition to the changes made to the manuscript.

Reviewer #5

(Remarks to the Author)
I co-reviewed this manuscript with one of the reviewers who provided the listed reports. This is part of the Nature Communications initiative to facilitate training in peer review and to provide appropriate recognition for Early Career Researchers who co-review manuscripts.

Open Access This Peer Review File is licensed under a Creative Commons Attribution 4.0 International License, which permits use, sharing, adaptation, distribution and reproduction in any medium or format, as long as you give appropriate credit to the original author(s) and the source, provide a link to the Creative Commons license, and indicate if changes were made.
In cases where reviewers are anonymous, credit should be given to 'Anonymous Referee' and the source.
The images or other third party material in this Peer Review File are included in the article's Creative Commons license, unless indicated otherwise in a credit line to the material. If material is not included in the article's Creative Commons

license and your intended use is not permitted by statutory regulation or exceeds the permitted use, you will need to obtain permission directly from the copyright holder.
To view a copy of this license, visit https://creativecommons.org/licenses/by/4.0/

Responses to reviewer comments on "Impact of two decades of intermittent ice shelf regrounding on the dynamics of Pine Island Glacier, Antarctica"

We would like to thank the reviewers very much for the time and effort they have spent reviewing this article. We have made many changes as a consequence of their detailed comments, resulting in a much clearer and more publication-ready article.

This document contains a reproduction of each review and our responses.

We have implemented the vast majority of changes suggested by reviewers 1 and 3, and a number of those suggested by reviewer 2. Where we have not implemented a suggestion, we have provided justification.

The reviewers all shared concerns regarding the style and clarity of the writing, so we have made substantial efforts to improve this – restructuring and rewriting large parts of the manuscript. (As a consequence of moving things around, the difftex document might contain errors or be difficult to interpret.) The article now follows a more conventional format (introduction, results, discussion). We have also modified the framing of the results relating to the role of regrounding in encouraging rift formation, to make it very clear what our observations show, what we infer from them and why. We have included a more detailed analysis of the elastic beam model to show why we are particularly interested in tidal hinging. We have made it clearer that our observations show that the rifts appeared in hinge zones surrounding grounded ice more explicitly in section 4.

It is also clear from the reviews that the methods section was inadequate in conveying our approach without ambiguity. For example, Reviewer 2 raised a number of high-level concerns with the manuscript, though these appear to stem from a misunderstanding of the methods rather than issues with the approach. The core question is whether intermittent regrounding measurably altered the stress regime of Pine Island Glacier's ice shelf (and hence PIG more widely), and the inverse and diagnostic modelling approaches we used are widely accepted as appropriate for answering such questions and remain well-suited to doing so. Hence, we have made edits to the methods section to better convey our approach.

We have included two new figures: figure 5 in the main section and figure 8 in the methods. We have changed figure 1 such that it shows where and when regrounding occurred relative to the bathymetry as well showing the remapped ice columns.

Below are each reviewer's comments and our responses. Our responses are coloured in blue.

Reviewer #1

This manuscript investigates the regrounding of the Pine Island Glacier (PIG) ice shelf on bathymetric highs and its influence on the mechanical stability of the ice shelf. My interpretation of the argument is as follows:
- Grounding on bathymetric highs occurs due to heterogeneities in the bed at the grounding line and in basal melt rates, producing thickness variations that propagate downstream.
- However, neither historic surface velocity data nor ice sheet modelling provide evidence that such regrounding increases buttressing; basal shear stresses in the newly grounded areas do not appear to increase.
- Instead, contact with bathymetric highs appears to promote increased rifting, driven by ice shelf hinging in response to tidal motion, which in turn leads to elevated calving rates.
- Previous numerical modelling study suggests that this calving contributed to dynamic ice loss from PIG between 2017 and 2020.

That is an excellent account of the argument.

Overall, the study is a valuable contribution, particularly in highlighting the perhaps counterintuitive result that regrounding does not necessarily enhance mechanical "stability" and may even have destabilising effects through tidal hinging, and I would recommend publication in Nature Communcations. The manuscript is generally clear, well-written, and the analyses appropriate for addressing the central aim.

We thank the reviewer very much for this assessment of the work.

My main observation is that the manuscript contains two key conclusions: (1) regrounding does not necessarily reinforce the mechanical stability of the ice shelf; and (2) regrounding can potentially lead to mechanical weakening. The first point is well-supported; the second (on the role of tidal hinging in driving calving) is presented as a key mechanism but is more speculative. The robustness of this mechanism is not fully established, leaving me uncertain about its role relative to other processes (particularly given the final paragraph in section 4.1). This is definitely an important mechanism to highlight – and if tidal hinging is truly responsible for the development of rifts and ultimate calving of PIG (which seems reasonable to conclude), then the modelling community needs to put more effort into capturing the dynamics correctly.

This is a very insightful comment, thank you. Indeed, the conclusions about the role of the regrounding on promoting rift formation rely on more circumstantial evidence than those about the regrounding causing minimal drag. As a reminder, the evidence is that rifting appears to occur near parts of the ice shelf that have regrounded, and that rifts appear in the wake of the bathymetric high-point. The inclusion of the paragraph about tidal hinging is meant to show that there exist physical mechanisms that would explain this, so that the more general hypothesis "regrounding was important for rift formation"

can even get off the ground. We have added much to the revised manuscript to clarify this and hope we have done so to the reviewer's satisfaction.

However, I would recommend tightening the manuscript's focus on the first conclusion: that regrounding does not necessarily strengthen the ice shelf mechanically. Where the mechanism of tidal hinging is discussed (particularly in Sections 4.1 and 5), it should be clearly framed as a potential explanation for observed calving rather than a conclusive driver. And I'd strongly recommend that section 5, in particular, be revised to reduce vague and speculative language (e.g., "intuitive," "natural to assume," "suggests… this is optimistic", "We leave these details to a future in which we can simulate the full viscoelastic behaviour of the ice shelf", "accurate prediction of future mass loss from the Antarctic Ice Sheet demands more subtle numerical modelling than previously thought"), which currently dilute the manuscript's main message. Clarifying these points will help convey the main finding from this study: that regrounding does not necessarily mechanically strengthen ice shelves.

Thank you for the useful comments. We have restructured things in this part of the manuscript so it is clearer that the results around rift formation are suggestive rather than conclusive, and that tidal hinging is a mechanism that could account for the observations. As per another reviewer's request, we have also made the tidal hinging argument slightly more concrete with the addition of a new figure (Figure 5). Regarding the second point around vague and speculative language, we have endeavoured to remove this entirely from the manuscript – thank you for the suggestion.

I otherwise had only a few specific comments:

- The final two paragraphs of the introduction could be a bit tighter with wording to improve clarity. E.g. at L46 "…reflecting changes to the bed topography at the GL as it migrates" – I originally interpreted this to mean landscape evolution, but do you mean spatial heterogeneity in the bed topography and how it influences the ice thickness? At L46-55, I recommend to precisely define "unsteady" and "stable/unstable" where used (i.e. for unsteady do you mean that it's changing rapidly? For stability/stabilising do you mean mathematical stability of the grounding line or the propensity for large-scale, rapid retreat).

Thank you for this comment. Looking back at this paragraph, it could be a lot clearer! You are right with the second interpretation of line 46. We have rephrased things here to make things clearer.
The use of "unsteady" is meant to reflect the common definition of "changing with time", while the use of "stable" in the final line of the paragraph is not in fact meant to reflect the mathematical definition that relates to the robustness of steady states to perturbations. We have altered the wording to be less ambiguous.

- Introduction: you could also cite Reed et al. (2024) who use a numerical ice sheet model to investigate evidence of marine ice sheet instability during past retreat events of PIG

Thank you for pointing out this omission, we have added this very relevant citation.

- L173: "…due to tidal motion in the vicinity of the grounded locations". Is it possible to

be more precise here – “downstream of the grounded locations”? Also could potentially link this to where we expect passive vs active ice regions of the ice shelf?
Good point. This section of the manuscript has been substantially rewritten and hopefully is less ambiguous.

- Section 6.2. How sensitive are the results to the regularisation parameters? Is 500 m sufficiently fine resolution to capture variations in the basal shear stress? I also wondered about the SSA equations and whether some of the other components of the stress tensor not resolved might be relatively more important where you reground.
It is a good point regarding the regularisation parameters. The answer is that the key features of the basal stress field recovered by the inverse problem (low basal stress, no localisation around the bathymetric high point) are very insensitive to the regularisation parameter in the central shelf. As noted in the manuscript, we used a particularly low regularisation strength compared to usual (in the BISICLES community) to give the model the best chance of displaying spatially or temporally localised changes in basal traction.
We consider 500 m to be sufficient resolution to capture variations in the basal shear stress. With the non-zero Tikhonov regularisation, it is definitely right to wonder whether grounding covering a single cell would be captured by the model, but at 500 m resolution, the observed areas of grounding generally cover at least 10 and often over 100 mesh cells.
The SSA comment is also a good one. Ultimately, we are not ruling out the importance of vertical shear in the evolution of the glacier, but think that this likely comes from tidal hinging rather than friction at the bed. If the friction at the bed is small, we wouldn’t capture the vertical shear with a full-stokes model anyway as there would be nothing to generate those additional stresses. We consider the SSA sufficient to answer the question of whether there is any signal in the ice velocity data indicative of regrounding as it is likely to be the horizontal stresses most impacted. For example, for the friction to cause any large vertical shear stresses (which might be generated to conserve angular momentum) the stress would have to be quite big, in which case it would be noticeable in the vertically-integrated horizontal stresses too.

Reference
Reed et al. (2024), doi: 10.1038/s41558-023-01887-y

Reviewer #3

Reviewer #3 (Remarks to the Author):

Surawy-Stephney et al., present a holistic study looking at the effects on regrounding at Pine Island Glacier. This work is a potentially important contribution as the influence of intermittent regrounding of an ice shelf is a fairly novel topic but there are some concerns that need to be addressed. Whilst I think the article provides an interesting study, I do not think it is best suited to Nature Communications.

We thank you for the positive assessment of the manuscript as a whole. Though it is ultimately up to the editorial team, we are of the opinion that the revised version of the manuscript is well suited to the Nature Communications given that the journal admits a longer format than others in the Nature portfolio. The rewrites and structural changes have given a bit more space to the different arguments presented in the manuscript which, we hope the reviewer agrees, has addressed this concern.

Overall, the writing style of the article could be improved. There are often clunky and difficult to read sentences and numerous references to demonstrative pronouns where it is unclear what the object is. I have pointed out some but there are lots of sentences that could be streamlined by removing unwanted parenthesis. I only point this out as some of the English detracts from the scientific messages.

Thank you very much for the comment. A lack of clarity in the writing was raised by other reviewers too, and we have rewritten large parts of the manuscript in an effort to improve this. The comment regarding demonstrative pronouns is particularly helpful in identifying where things weren't making sense – thank you for the clear instruction. We hope that the revised manuscript addresses this concern.

I have several major concerns that I outline below:

1. Methods. It is unclear if imposed grounding is only on the diagnostic simulations or the inversions too. Reading the methods it seems clear that the imposed grounding ice is only for diagnostic simulations but in Section 3 of the main text it is unclear. I suspect this is due to a lack of distinction between 'imposed grounding' used in the diagnostic simulations and 'natural grounding' which occurred during the observed time of the inversions. It would help greatly if the authors could add some clarity to the text and add a couple of sentences defining each.

This is a very good point. We imposed grounding over the full ice shelf during the inverse problems used for inference about the basal stress field, and grounding only in the location of the bathymetric high-point during the diagnostic simulations. Note also that the diagnostic simulations were initialised by solving inverse problems, but that these are not the same as those used for inferring potentially non-zero basal stress in the region of the bathymetric high-point. We have edited the results and methods sections to disambiguate these things, and hope the reviewer finds the resulting description of our approach clearer.

Diagnostic simulations – I am not opposed to the methods of moving the bedrock even on a real domain but it would it be good if the authors could explain the reasoning behind this i.e. explain why you don't just thicken the ice shelf over the grounded and near grounded area.

This is a good point, thank you. The idea is to add basal stress without modifying the driving stress, to try and isolate the effect that the grounding of a section of the ice shelf might have on its dynamics. Thickening the ice would modify the driving stress. That could be a reasonable strategy if we were confident in the bed topography under the ice shelf, but it is very approximate, so thickening the ice to come into contact with it would bring in too many confounding factors. By elevating the bedrock, we can add a term to the stress balance (the non-zero basal friction) while leaving everything else the same. We have added a sentence to the methods explaining this.

It took me a couple of read throughs to understand the modelling methodology. As I understand –

1) Invert for C (ungrounded ice has no basal traction). Used for inversion results.
2) Raise bedrock over imposed area.
3) Prescribe values of C on the imposed grounded ice related to the basal stress from the Joughin et al., (2019) regularised Coulomb function.

Assuming the above is correct, the diagnostic simulations sections should be improved for clarity. The main text also confuses the order of the methodology and separation of the inverse and diagnostic simulations.

That is indeed correct. We have rewritten the results and methods to make it clearer what we are doing – we hope the reviewer finds the explanation more thorough.

2. The role of regrounding in calving between 2015-2020. It is unclear what this section adds to the main manuscript. It is mainly a description of observations of rift formation of the area of regrounding. The descriptions seem reasonable, but it is vague it how these rifts produce calving. I suggest either the subsection title is changed to something like, 'The role of regrounding in rift formation' or more detail is added as to how/if these rifts produce calving. For example, what is the time for advection from the area of grounding to shelf wide rifting? Can the calving waiting times be linked to the timing of the regrounding?

These are all very good questions – thank you for raising them. This is the part of the manuscript we have changed most substantially. We have made it clearer what our results show (as the reviewer says, that rifts form in the area of regrounding) and why this might be. We hope the new version of the article does a better job at motivating the questions we discuss.

To answer the specific questions, the rifts that we highlight in figure 4 with the red ellipses all go on to propagate to the margins and produce large, tabular icebergs. We do not see additional rifts appearing between these that do not go calve icebergs. However, we did not move beyond this analysis to look at how long it took for rifts to

propagate to the margins etc. as it is not clear what we would learn from this without understanding the physics of crack propagation a bit more completely.
We have taken on board the point that our results are not as definitive as the writing sometimes made out, but we hope the framing in the new version of the manuscript addresses this. For example, we have included some general discussion of the likely causes of the calving and where regrounding might fit in (lines 143 onward in the new manuscript).
I have considered performing a more thorough analysis of what caused the calving on the ice shelf. This would involve performing a series of inverse problems going back further in time, accounting for terminus retreat, shear margin retreat and shear margin weakening to quantify stress changes on the central shelf. However, we would be left with the same question as to what seeded rifts in the central shelf that allowed such a short period of rapid calving. We consider such additional analysis beyond the scope of this article and leave it to future work.

Furthermore, a simple analysis of the 2D planar stress/strain field (presumably available from the BISICLES simulations with imposed grounding) could provide insights into rift propagation. Similar to the authors comments about the basal stress the magnitude is of secondary interest and instead the ice shelf stress pattern is important. Looking at Fig. 3a panel 4 I would be surprised if the stress contours did not replicate the observed rifting seen in Section 4. Such analysis could add weight to the study in terms of ice sheet calving. All calving functions, whether damage or stress-based, rely on a SSA-style stress field. If the viscous dynamics do not pick up any changes in the stress field then the authors arguments on tidal hinging become more potent.
This is a fair comment. There is nothing in the ice speed data that show any sharp increases in longitudinal stress that could cause rift formation. There are, of course, larger strain-rates over the rifts once they form, but we cannot conclude anything about the causes of their formation from these. As stated in the new version of the manuscript, it is very likely that shear margin retreat and weakening led to a general slow redistribution of stress from the shear margins to the central trunk. Ideally, were we to analyse this change in background stress, we would perform inverse problems going back to the time in which Pine Island Ice Shelf was joined at its northern margin to its neighbouring ice shelf (the mid 2000s). We would then compute stresses as the shear margins retreated and weakened. However, this would require a lot more work and is beyond the scope of this article. (Especially as the resulting increase in longitudinal stress on the central shelf seems so probable!) The result of this is an ice shelf where stresses are highest in line with the edges of the shear margins, so you would expect calving there. However, the huge increase in calving rate requires explanation beyond this. That is where we argue for seeding of cracks (or growth of existing ones) due to regrounding.

3. Although I agree with the authors that tidal hinging is a potential source of failure, it is hard to justify the extent to which they have taken the argument without convincing modelling study. I would suggest this is instead phrased as a potential implication of the inversion/diagnostic simulations or a more detailed modelling study is done.

This is a very fair assessment. We have made slightly more of the elastic beam model as a rough indication of additional stresses placed on ice as a consequence of flexure, but we take the point that a convincing modelling study is still required. Hence, we have changed the framing of this part of the study to reflect its more speculative nature. Hopefully this comes across in the revised manuscript.

4. I think this is a really interesting study that the Nature format does not do justice to, particularly with regards to the methods. Additionally, a longer format article would allow the authors to expand their study in relation to calving which at the minute is purely speculative.
We thank the reviewer very much for taking an interest in the study. Given that Nature Communications does admit longer format articles, we think it is an appropriate journal choice though, of course, leave the decision with the editor. We hope that the newly written manuscript gives enough space to the arguments while remaining an appropriate length and style for Nature Communications. We agree that the results regarding rift formation and calving are supported by less direct evidence than those regarding basal friction, however, disagree that they are purely speculative. As stated above, in an ideal world we would perform more analyses of how longitudinal stresses changed as a result of shear margin retreat and weakening. However, we don't consider this too great an omission as the change in calving style over a short period is so dramatic as to warrant further explanation anyway. (Note that there have been no calving events since 2020 despite the shear margins being now at their most retreated positions). We show that the crevasses appear in the hinge zones of regions of ice that have grounded and, while this is not definitive, we consider it evidence in favour of the hypothesis that regrounding had a role in rift formation and, therefore, calving.

5. The nice modelling and observation results of the paper indicate regrounding does significantly impact ice velocity. This is an interesting result but it is not presented as the main conclusion. Instead the focus is on rift formation from ice shelf regrounding which comparatively less evidence is provided for.
Thank you for the comment. We have revised the manuscript so that the framing of the results is more in line with the strength of evidence we have, and hope the reviewer finds this more appropriate!

Minor Comments.

Ln 31 what 'things'?
We have altered this line to read "The flow speed of the glacier increased with each stage of grounding line retreat during this period".

Ln 44 'subverting' not sure this is the best term.

We have changed this to “a departure from”.

Ln 46 ‘like this’ what is ‘this’ referring to?
“like this” was supposed to refer to the description of PIG in the last two paragraphs. However, we agree this is ambiguous, so have changed this to “A glacier undergoing acceleration and grounding line retreat…”

Ln 49 Stylist. The sentence could be restructured to avoid back-to-back citations.
We have changed this sentence round to avoid back-to-back citations.

Ln 53 Rephrase. This is a difficult sentence to follow.
We have rephrased this and hope it is clearer now.

Ln 110 It is unclear if ‘fictitious’ stress refers to any basal stress on the ice shelf or only in the imposed regrounded area. Or is this purely referring to whether the inversion indicts regrounding based on the spatial pattern of the basal stress prior to imposing the regrounding?
This is a very good point and indicates some lack of clarity in the methods which, hopefully, the new manuscript addresses. This is referring to *any* basal stress inferred over the ice we know to be floating in reality, but we have grounded in the experiment. For this particular experiment, we have grounded the whole ice shelf.

Ln 135-137 This sentence is confusing and needs to be clarified. I think the full stop is also meant to be a comma.
Thank you for the comment. We have rephrased this paragraph.

Ln 301 states the use of ‘time-evolving calving front positions’. Are these monthly constrained i.e. one front position for each monthly inversion or are you evolving the geometry whilst inverting? Ln 104 in the main text would suggest the terminus evolving.
This is a good question. We are prescribing the calving front boundary only when a calving event occurs, and do not have the model advect ice downstream to advance the calving front. The reason for doing so is that small deviations in terminus position between updates have negligible influence on the recovered basal stress in the central shelf—the region of interest. In particular, the advances of the calving front seen on Pine Island Ice Shelf between timesteps proceeded without accompanying advance of the shear margins, and this unconfined portion of the ice shelf does little to impact the upstream stress.

Ln 306 how much do you have to raise the bedrock for each simulation? Is it within tidal range or does it rely or a much greater ice thickness shift? This would allow the reader to get a sense how likely regrounding is.
Thank you for the good question. The answer is around 150m (and the ice shelf thickness is around 550m). However, it is not worth including this in the article as it gives the impression that we have confidence in the bathymetry in the region. The bedrock elevation is almost certainly higher in reality than the bathymetry suggests. For example, the ice shelf regrounds on a place where the bathymetry “data” actually suggest quite a large dip in elevation.

Ln 314 I would advise against using 'fictitious grounding region' perhaps imposed grounding region?
This is a very good suggestion which we have implemented, thank you.

Ln 316 This sentence is awkward and difficult to understand.
Thank you for pointing this out. We have changed the sentence from "The choice of C changes the speed, so the stresses computed using eq. (4) after the problem has been solved were reduced compared to these." To "Of course, the choice of C changes the speed that the model predicts. Therefore, the stresses that the ice experiences differ compared to the target stresses."

It would also be useful for the authors to establish why they prescribed the friction on imposed grounded area rather than inverting.
This is a good point, thank you. The reason for doing this is to show the kinds of variations in the timeseries of observed ice speed we might expect to see if the grounding *did* impart some basal stress. Simply grounding a small part of the ice shelf in the centre, inverting for basal friction there and trusting that the answer is a good reflection of the true basal stress is putting too much faith in the inverse problem, which we know to be ill-posed. (This can be seen in the fact that the recovered basal stress across the ice shelf is non-zero in the experiment in which we ground the ice shelf.) Instead, we have opted for a slightly more complicated set of methods, but one that intends to bias the results less. Hopefully the set of methods as presented in the revised manuscript seems sufficient.

Ln 322 Stylist. This could be rephrased to avoid the references backing into each other.
Thank you, we have rearranged the sentence as suggested.

Figures. Larger text on axes.
Thank you for the comment, we have made the axis labels larger.

Reviewer #2

GENERAL COMMENTS:

This paper is about the numerical modeling results of Pine Island Glacier's regionalized basal drag at a bathymetric high in the ice shelf and whether the grounding that has taken place in the past over this bathymetric high has had any impact on the ice shelf's dynamics. The paper does not feel fully developed and is difficult to follow at times. I highlight both of these concerns in the following paragraphs and in the line comments. At this time, I do not recommend that this paper be accepted for publication.

We thank the reviewer for taking the time to provide an extensive review of the article with many useful comments. We have made extensive efforts to rewrite the manuscript in line with these comments. However, it is clear there has been a failure of communication on our part which has led to some misunderstandings about the work. Hence, some of the comments are a bit difficult to interpret and, hence, respond to in a constructive way. We hope that the reviewer finds the changes we have made to the article have clarified our approach and framed the results in an appropriate way.

It is incredibly important that the authors stress the differences between the ice sheet's grounding line and the ice shelf's ice rise grounding line. The use of the word "regrounded" is often ambiguous to the various spatial grounding lines referred to throughout the abstract and the entire manuscript. The authors need to very obviously emphasize that the "regrounding" is in reference to the ice shelf's ice rise and at no point in the manuscript do the authors touch on any changes to the ice sheet's grounding line. In the detailed comments before, I have highlighted every instance where it is unclear what part of the ice shelf is being "regrounded". Additionally, the authors need to provide a definition of "regrounding" and "grounding" relative to the manuscript because it is not clear if either term is a fixed state, temporary, or reoccurring. This is especially true with the comment made on Line 70 about "ephemeral" grounding.

Thank you for the useful comment, we have updated the manuscript to define what we mean by regrounding (in the abstract) and now use that term throughout.

The data being presented in the manuscript's current state makes the implied conclusion that the ice shelf's ice rise role in PIG's grounded ice dynamics an extreme reach.

It is difficult to know what conclusions the reviewer thinks are a reach, but assuming that they are referring to *any* conclusions about the effect of grounding on the bathymetric peak on dynamics, we respectfully disagree. The methods we use are sufficient to answer such questions and do so, at least in the case of the effect on the horizontal stress balance, pretty unambiguously. The new version of the manuscript makes it even clearer where we consider our conclusions to be robust under the evidence provided and where we are speculating. To assess the direct effect on dynamics through the addition of basal stress, we make use of widely used inverse methods to extract information

about the time-evolving stress balance of the ice shelf, and separately compared diagnostic simulations of ice velocity with observations. This comment perhaps points to a misunderstanding of our approach. To clarify, we did the following things:

Experiment 1:
Performed inverse problems over the ice shelf pretending it was fully grounded. The reason for doing this is to see whether there is anything in the ice velocity data (from Sentinel-1) that suggests localised basal traction in the region of the bathymetric high point, when we know grounding occurred there. The inverse problems were performed for every month of ice velocity data we had available. The results of the inverse problems suggest a smooth field of low basal traction across the ice shelf (stopping short of the true value of zero – a feature of the ill-posedness of the inverse problem) that doesn't vary much in time or localise in space around the bathymetric high point. The conclusion is that there is nothing much in the velocity data that suggest there is more likely to be basal traction around the bathymetric high point than anywhere else. (One could extract some information from the velocity data directly, though a greater set of assumptions would be required.) Then we moved onto experiment 2, to check the degree to which regrounding would be visible in the ice timeseries of velocity data if it imparted nonzero basal stress:

Experiment 2:
Step 1: Perform inverse problem over the whole PIG to initialise the simulations. Note, these are different from the inverse problems performed in Experiment 1 and the results are not discussed in the article as they are not relevant.
Step 2: Add small amounts of basal traction in an isolated region of the central shelf and recalculate ice speed.
Step 3: Compare changes in modelled ice speed with different amounts of basal traction to fluctuations in the timeseries of observed ice speed.

Hopefully this clarifies what was going on. We have adjusted the methods section of the article in an attempt to clarify these things.

I understand the thought process behind why basal drag was modeled, but there are several other means of assessing the bathymetric high's effects on the ice shelf that rely primarily on actual data, so I wonder if the methods implemented in this project are overly complicated and considering the amount of smoothing that seemed to have been applied to the data, maybe alternative assessments would be more impactful and accurate in the final analysis. It seems that despite there being observational data available from a multitude of sources over the timeline of the study, that what the authors have done is model basal drag from modeled or "idealized" coefficients and then used that value of basal drag to model the surface velocity for valuating the impact of the bathymetric high. Considering there is multitemporal velocity, elevation (e.g., REMA, ASTER, OIB), and some ice thickness—which can be used for validating hydrostatic thicknesses from surface elevations—datasets, why wasn't a more direct means of estimating basal drag like van der Veen and Whillans (1989) or MacAyeal (1993) used?

Lines 107-112 leave the reader doubting how realistic the modeled results are. More importantly, why was only basal drag presented? The stress with the greatest impact on rift propagation is lateral shear. Why wasn't shear stress, or at bear minimum, shear strain, calculated/presented in the results? What about surface elevation changes of the ice shelf? There should be plenty of REMA data over the timeline of this study and you already have surface velocities, so calculating Lagrangian vertical changes in ice thickness should be relatively straight forward. The heigh above buoyancy over the bathymetric should really should be calculated at each grounding time step (or with a temporal reference that is as close to the InSAR data as possible) as another means of supporting the argument that grounding is taking place.

This again suggests that we haven't been clear enough with our description of the methods. For example, the reviewer writes the following sentence: "what the authors have done is model basal drag from modeled or "idealized" coefficients and then used that value of basal drag to model the surface velocity for valuating the impact of the bathymetric high". It is difficult to understand what this means or how it relates to what we have done, but hopefully the explanation above and the newly written methods section make things clearer.

It is suggested that we have performed lots of smoothing on the data we use - presumably a reference to the ice speeds we use in the inverse problem misfit? When the ice velocity data is produced, there is indeed some smoothing. For example, the size of the windows over which features in the backscatter data are tracked are large and overlap, which imparts some smoothness on the velocity field. Additionally, creating monthly mosaics from velocity fields calculated from individual satellite acquisition pairs is a smoothing step. But we do not perform any smoothing ourselves in addition to this standard practise by the generators of the dataset, and the level of smoothing is not so great as to destroy the signal we are interested in. Note, for example, figure 3, which shows that the effect of the non-zero basal traction is distributed widely across the shelf.

The next suggestion of the reviewer is that we ought to have looked at lateral shear stresses in our assessment of the effect of the grounding on the dynamics. However, through the use of the numerical inverse methods, we account for *all* the components of the horizontal stress. This is a very good reason to use this kind of method of inference: so we don't have to rely on much less well-motivated methods for extracting stress components from maps of ice velocity.

The reviewer then recommends using the methods of MacAyeal (1993) to directly estimate basal drag. This is indeed exactly the method we use. It is reassuring to know that we agree that this approach is sensible, even if we were not able to communicate it in the article.

The idea of using REMA DEMs to create Lagrangian thickness change fields is a good one, though the reason given to do so given above is in order to provide additional evidence that the ice shelf grounded on the bathymetric high-point. However, the evidence from the differential range-offset tracking is very strong, such that this addition is not required. For example, see Joughin et al., 2016, Wallis et al., 2023.

I am confused about what exactly is being used for parameters in the BISICLES (Cornford et al., 2013) algorithm. In solving for basal drag, surface elevation, surface slope, and ice thickness are required inputs. Based on the provided text, it seems that those parameters only come from BedMachine v3 (Morlighem et al., 2022)—is this the case? If so, then the authors need to provide something of support either in the form of additional model runs or from the literature to show that using a temporally static dataset still produces accurate basal drag results. According to Bevan et al, (2023), using a constant BedMachine ice thickness does result in reduced ice velocities in temporally dynamic model runs. As that study represents a timeline of modeled results significantly longer than this one, I do not know what the full impact using a temporally static dataset will have on the accuracy of this study's results.

The bed topography comes from BedMachine v3, yes. The temperature field is produced according to a thermomechanical spin-up. The surface elevation is according to REMA. We produce calving fronts by annotating Sentinel-1 backscatter images. The argument in Bevan et al., 2023 relates to the use of BedMachine v2 (different to the data we use) in time-evolving prognostic simulations. They find that, during the inverse problem, that thickness product produces driving stresses at the grounding line during that are too large. Hence, the basal friction coefficient that they infer from the ice speed data is too large. Some timesteps later, the relaxed thickness combined with the large basal friction coefficient results in unrealistically slow-flowing ice. So they relax the thickness during the inverse problem to solve the issue. This is standard practise in initialising prognostic simulations, though is not relevant to our study. We are interested only in inverse problems and diagnostic simulations where these considerations are much less important. For example, it doesn't make a difference whether the driving stress at the grounding line is slightly over-estimated, as the inverse problem will produce a basal traction field there that gives us the right stresses in the place we're interested in, namely the central shelf. The more relevant point revealed by Bevan et al., 2023 is how important the use of a retreating calving front is for the stresses on the shelf. We heed that lesson here, by ensuring that the calving front we use is appropriate for any time period covered by one of our inverse problems. So, to answer the reviewer's query: the use of a temporally static dataset will have little impact on the accuracy of this study's results.

I would like to know why the ice sheet's grounding line was not estimated along with the bathymetric high's grounding line. Surely the location of that major boundary is playing a roll in the grounding of down-flow, isolated grounding. I may be wrong, from the Rignot et al, (2016) grounding lines, it looks like the 2011 grounding line is in use for the numerical modeling. What evidence do the authors have to support using a temporally static boundary for the basal drag estimates?

We thank the reviewer for the interesting comment again. The location of the grounding line is certainly important for the thickness of the shelf at different locations. However, the parts of the shelf that are of interest passed over the grounding line before our observations of ice speed and thickness began so it's not easy to perform that kind of analysis. Thankfully, it's also not relevant to the questions we pose in the article. It would

be very interesting to see a study on the historical changes of ice thickness at the grounding line (requiring a long timeseries of grounding line location and surface elevation or surface elevation change data) and how this relates to downstream changes, but we are not willing to undergo that analysis here. Regarding the kind of modelling performed in the article, it is acceptable for the grounding line to be a kilometre or so out of place as it can be compensated for by only small differences in the basal traction.

In Sections 2-4, both results and discussion points are included in each section. It would be better to parse results from discussion points because the discussion points are written very passively and it takes away from impact of the results.

Thank you for the comment. We have heeded this advice and restructured the article into a more standard format to distinguish our results from more speculative discussion points.

LINE COMMENTS:

Line 4: It is unclear here what exactly is being "regrounded" if the authors are referring to the ice sheet grounding line or the ice shelf ice rise grounding line.

We have added a sentence to the abstract that clarifies this here. We hope the reviewer finds this less ambiguous throughout the rest of the article.

Line 5: Refer to a general "hypothesis" in the previous sentence before using "this hypothesis" in this one.

We are of the opinion that we can introduce "hypothesis" here and it be unambiguous to what we are referring.

Line 8: "that this had on the dynamics of the glacier"—this is a misleading statement. The only results that illustrate any form of generated flow assessment is Figure 3a and that barely shows any of the grounded ice. It also seems like there is a much better way of highlighting the secondary influences rather than referring to them as "indirect".

Even though we don't show "generated flow assessments", we do analyse the effects on the dynamics of the glacier. We find that the regrounding had no effect through the introduction of basal traction, but that it might have had an effect through the promotion of calving. Both of those possibilities would be linked to dynamics at the glacier scale.

Lines 9-10: "regrounding"—regrounding of what? "it likely contributed to the

promotion"—there was virtually no evidence at all to support this statement. Saying something less definitive like, "one potential driver behind the 2015-2020 calving was the possible..." is more reflective of the presented results. "held largely responsible"—what was? It's probably in reference to "regrounding" but it's not obviously that that is the case.

We have changed "likely" to "plausibly" and reworded the sentence to make it clear that it is the calving that is held responsible for the dynamic changes.

Line 11: "regrounding"—of what? "systems"—way too broad of a term. Be more precise and use phrasing specific to glaciology/marine terminating glaciers/ice shelves/etc.

Thank you for pointing out this ambiguity. We have changed this to "glaciers".

Line 14: Making a bold statement that PIG is "currently" losing the most mass of any glacier in the world needs to have a much more up to date reference than the 2017 data used in Rignot et al, (2019). Additionally, double check that in the last few years Thwaites hasn't bypassed PIG for that title of most mass loss.

Thank you for the fair comment. Given that the statement is quite dependent on timescales, perhaps it's best to not to be superlative about it. We have changed this to the safer statement of "Pine Island Glacier (PIG) experiences one of the highest rates of mass loss in Antarctica" and kept the same reference.

Lines 20-22: This sentence needs a reference, or say something along the lines that the authors have observed said changes. "The early stages of the retreat are difficult to characterise"—why? If due to a lack of observations, then say that. Also, include an approximate range of time when the retreat began.

Thank you. We have incorporated both of these comments.

Lines 23-25: "perhaps aided by a lack of available intermediate steady-state grounding line positions"—what does this mean?

It means that there might not have been any steady state grounding line positions, given the forcing, between its position on the bathymetric ridge and its current position. It's a statement about marine ice sheet instability. But it's still somewhat debated, hence the "perhaps".

Line 24: Delete the 's' from "shows".

The 's' should be there as "imagery" is a singular noun.

Lines 26-28: During those dates provided, Joughin et al, (2016) reports that this is when the island formed and not the peninsula.

Thank you very much for pointing out this mistake in the timeline. It has now been rectified.

Line 30: “this ice keel”—up to this point, a specific ice keel has not been referred to, so “this ice keel” is confusing.

Thank you for pointing out this ambiguity, we have addressed this in the revised version of the manuscript.

Lines 31-45: The background information is useful, but it is misleading for the reader because all mentions of the grounding line are with respect to the ice sheet grounding line and has nothing to do with the ice shelf ice rise grounding line. Considering the direction of the paper, this paragraph should only be referencing facts about the ice shelf and nothing to do with the ice stream or the ice sheet grounding line.

The reason for providing this background on the main grounding line is that the article is about dynamic changes on the glacier as a whole. Given the article’s interest in calving, we feel that it is important to set out the idea that there have been at least two phases of dynamic change in the observational record. The first is characterised by a retreat of the main grounding line and an accompanying increase in flow speeds. The second by a retreat of the calving front and an accompanying increase in flow speeds. The article goes on to analyse the impact on the dynamics of the glacier of grounding of parts of the ice shelf on the bathymetric high point. It is necessary to have the background of what people think has driven dynamic change in order for the suggestion that the grounding influenced calving behaviour to be of interest.

Lines 46-55: Like the previous paragraph, the references to the ice sheet’s grounding line are confusing because this study literally does not include a single bespoke observation of PIG’s grounding line. Ice sheet grounding line inferences need to be removed. Details about past observations of changes to PIG’s pinning points or shear stresses are actually relevant to this study.

Hopefully the new introduction makes things less ambiguous. We feel that, as the reviewer suggested, defining “regrounding” more clearly early on makes it obvious that we have no interest in what the main grounding line is doing so the introduction doesn’t lead readers in the wrong direction.

Lines 46-48: “system”—use a different word that is specific to ocean-terminating glaciology. “changes to the bed topography”—the bed topography is changing? Unless sediment deposits hundreds of meters thick are taking place, this seems unlikely. “different parts of the ice”—use more unambiguous language.

Thank you for pointing this vague wording out. In general, this part of the introduction could have been a lot clearer, so we have made changes to make it so.

Lines 49-51: Make sure to include that the Bindschadler reference is with respect to PIG and not glaciology as a whole. “the imprint on the fine structure of the thickness”—what

is this? If this is implying that past flow conditions of grounded ice can be observed from the surface of an ice shelf, then Hulbe and Fahnestock (2007) is a better source to cite. “grounding line geometry”—geometry of what? Glacial ice?

Thank you, we have clarified that the Bindschadler reference is with respect to PIG. We have rewritten this paragraph in a way which hopefully makes it less confusing.

Lines 51-52: “natural to assume that positive perturbation in the thickness”—this is confusing. Are there perturbations horizontally homologous? Is the areal coverage over the entire glacier, grounded, or floating ice? Short-lived increases in ice thickness at an ice sheet’s grounding line could actually be evidence of mass imbalances of ground ice flow increases causing greater amounts of grounding line ice discharge like what has been observed on the Crosson Ice Shelf (Mouginot et al., 2014).

We agree with these comments, thank you. We have made substantial revisions to this part of the manuscript to make it less confusing.

Line 60: These displacements vertical or horizontal?

These are measurements of displacement in the range direction.

Line 62: “would”—use more active language.

Thank you for the recommendation, we have removed the word “would”.

Lines 68-69: The citations are confusing. Did Kimura et al, (2016) produce the data in the state with which it was used in this study? If not, then there’s no point in citing that body of work here.

Yes, this is a good point. The data is basically that of Dutrieux et al., 2014, but modified with some data from a additional 2014 UAV campaign. The version of the bathymetry data used here is the same as that used in Kimurna et al, 2016. We have altered this sentence to make it clearer.

Line 70: “hot spot”—use a different phrase. Up to this point, there has been no evidence provided that shows the bathymetric high is a point of ice damage, so “hot spot” is confusing. “ephemeral”—so, how do the authors actually define what grounding is? How does this differ to “light grounding” referred to on Line 82.

We did not intend to invoke any idea of damage, so have changed the sentence around such that a “hot spot” is no longer mentioned. We define “grounding” as the state when ice touches the ground. This can be ephemeral or not. We have added to this paragraph to make it clearer what we mean by ephemeral and light grounding.

Lines 71-73: “flow velocity”—where does this data come from? “to keep track of which parts of the ice shelf have been grounded at different times…”—this is ambiguous language and too simple for a journal article. Concisely tell the reader that following the

ice shelf grounding experienced by those spatially discrete regions, remapping techniques were used to temporally track the ice columns to their present-day locations.

Thank you for the comment, we have made it clear in the revised manuscript that these flow velocities are satellite-derived, though leave it to the methods section to set out more completely the datasets involved. Thank you for suggesting a cleaner and more appropriate wording, we have changed the text to match this.

Line 73: In Figure 1's current state, it is untrue that regionally discrete, temporal ice shelf grounding is being showcased. See specific comments about the figure.

We have modified the figure to address this and hope the reviewer agrees it shows the history of contact between the ice shelf and the bedrock.

Line 75: With the reference to "thinning" here, were surface elevation changes assessed in this study? There are much more current studies (e.g., Davidson et al, 2023) using data newer than 10 years old as stronger evidence to support whether thinning has taken place.

Davison et al, 2023 look at basal melt rate rather than thinning rate. We also note that it is useful to have an estimate of the thinning rate on the ice shelf from near the centre of our timeseries of observed grounding rather than now.

Line 76: "This is also evidenced…"—what is also evidenced? 4-year consistent floating?

Thank you for pointing out the ambiguity, we have changed this sentence.

Line 85: "The methods we use"—too broad and passive. Say, "Our InSAR processing requires…"

Thank you, we have changed this to read "The DROT method requires…".

Line 86: "We emphasize the difference…" Where? How? Did I miss this somewhere?

The use of this phrase was not intended to indicate that we have previously made that point. However, we have changed the paragraph so as not to confuse people.

Line 89: "…observation that grounding appears to have occurred…"—passive language. Say, "…interpretation that grounding occurred…"

Thank you, we have removed "appears to have".

Lines 90-91: "…perhaps corresponding to periods of large tidal amplitude, or diminished mean sea levels due to regular oscillations in atmospheric pressure."—Do either of these parameters actually statistically correspond to the timeline of grounding? If not, this is both extremely passive language and irrelevant to the observations.

We have removed this part of the sentence altogether.

Line 94: “grounding”—grounding of what? “influence”—influence of what?

We have changed this sentence to read “The most direct way by which the regrounding of the central ice shelf could have exerted an influence on the dynamics of Pine Island Glacier is by changing the local balance of horizontal stresses through the addition of contact friction on the base of the ice, form drag at the bottom of the ice column, or vertical shear within a deformable bed.”

Lines 99-101: I am confused by this. Why is it necessary to “raise the bedrock”? If Schoof and Hindmarsh (2010)’s shallow shelf approximation is what is being implemented in the BISICLES, then this shouldn’t be necessary. It seems too that if the bedrock was “raised” to touch the bed of the ice shelf, then the surface relief will also need to be altered to mirror the bed geometry the ice is flowing over in which case driving stress and ultimately basal drag will also be affected. Cornford et al, (2015) does mention that some previous studies have modified the bedrock elevations so that ice thicknesses match discharge rates at the ice sheet grounding line, but this is not an equivalent scenario for the ice shelf bathymetric high. Additionally, it is not clear what the spatial location of where raising the bedrock took place.

We hope that the newly written methods section makes it clear what we have done. There is no sense in which doing so was “necessary” other than it was the experiment we chose to perform.

Line 102: “namely” is an awkward word choice.

We could change this to “specifically” or a similar word, but “namely” is the most precise and correct choice in this context.

Lines 102-103: How do the authors know that using nearly static terminus positions produced accurate results?

Inverse problems were solved using calving-front masks updated after each major calving event (2015, 2017, 2018, 2020), so the terminus position was not fully static. This approach ensures that the geometry reflects observed changes during the period of interest. Because our experiments are diagnostic rather than prognostic, small deviations in terminus position between updates have negligible influence on the recovered basal stress in the central shelf—the region of interest. In particular, the advances of the calving front seen on Pine Island Ice Shelf between timesteps proceeded without accompanying advance of the shear margins, and theory is clear that unconfined ice shelves do little to the upstream stress. We have added a sentence in the Methods stating this.

Line 127: “grounding during this period”—what exactly this is timeline?

Thank you for raising this ambiguity. We have noted that the timeline is October 2016 to December 2021.

Lines 130-132: This reads as though the BISICLES and thus the Shallow Shelf Approximation was the wrong choice of model for this study...

Just as a reminder, the line in question here is:
"This observation suggests that it is unwise to extrapolate basal stickiness coefficients to sub-shelf cavities when running simulations of marine ice sheets in which regrounding can occur, and highlights the merits of effective-pressure-dependent sliding laws."
This does not suggest BISICLES or the SSA are inappropriate for this study. We are making the point that under prognostic simulations in which ice can ground on areas of bedrock for which the stickiness has not been estimated using inverse methods, one should not simply extrapolate stickiness to those areas or, if that is the only method available, one should be cognisant of the fact that small isolated spots of grounding could exhibit low effective pressures at the ice base. We have moved this line into the new discussion section and hopefully made it a bit clearer.

Line 134: This section title reads as though an analysis is carried out, but that is not the case and no real conclusions about the "role of regrounding" are determined. This section needs to be renamed to reflect the purely observational nature of it.

Thank you for the comment. The section is now entitled "Regrounding and the development of rifts". We have changed the manuscript to make it clear what our observations show (that rifts formed in the hinge zones around areas of ice that made contact with the bedrock) and what we speculate given these observations. It was never our intention to over-emphasise our observations, and we hope the reviewer agrees that the language in the current version is fair and well-reasoned.

Line 135: Lines 126-127 directly contradict this. Additionally, "glacier dynamics" is not the same thing as ice shelf dynamics and should be altered. The authors have not modeled any of the grounded portion of PIG's stress regime to be able to make a comment on whether the glacier's dynamics have been altered.

We do not see a contradiction with Lines 126–127, as Line 135 is written in the conditional. We agree that we have not explicitly modelled the evolution of the grounded portion of PIG, but the dynamics of the grounded and floating parts are strongly coupled, so the distinction is not particularly meaningful. Perhaps the confusion arises as, potentially, some people use the word "glacier" to only refer to the grounded part? Perhaps the reviewer can comment on whether this is widespread and, if so, we can consider changing the manuscript to avoid ambiguity.

Lines 136-138: PIG is sensitive to changes in its lateral stress. To say that it is sensitive to changes in the terminus is misleading.

Thank you for this comment. We have removed this line. We have written a bit more background (around line 145 in the revised manuscript) that outlines what stresses PIG is sensitive to.

Line 139: “pattern of calving” is ambiguous. Refer either to the calving rate or the areal region of calving events.

We have changed this sentence to read “In contrast to the previous calving rate of around one iceberg per decade...”

Line 140-141: Replace “margin” with “terminus”.

Thank you, we have made this change.

Line 148: “this unfamiliar mechanism”—referring to the 2018 calving event, or the central origin of rifts? “usually associated with rift initiation at the ice margins”—the way this is written, it needs a reference. If it is instead more specific to PIG calving, then include something about how this is an observation of the authors.

Thank you for pointing out this ambiguity, we have adjusted this paragraph to be clearer, and included appropriate references.

Line 152: “We investigated the spatiotemporal coincidence of rifting”—in what sense? This part of the sentence made me think that geostatistical analyses were going to be conducted. It is more appropriate to simply say that observations were made. “with the grounding of ice”—what “ice” is being referred to here?

Figure 4a shows explicitly the spatiotemporal coincidence of rifting and regrounding. It is the ice shelf of Pine Island Glacier that is being referred to. This section has been fully rewritten with a focus on clarity so, hopefully, when read through, this kind of thing should now be unambiguous.

Line 158: Just because the rifts are first observable down-flow of the bathymetric high it does not necessarily mean that they are the result of a grounding event. Use "down-flow” instead of “wake” here and on Line 159. Particle tracking has been used to assess horizontal displacements of the grounded regions—it should be reasonable to assign a grounded event to a rift if the assumption is made that the rift’s initial fracture first propagated during the grounding event.

Thank you for the suggestion of not using “wake”, we have changed this to “downstream”. Yes, this is the conclusion that is being drawn. If the rifts occurred as a result of tidal hinging, then they should appear near to the regions of the ice shelf that regrounded, but not necessarily on top of them. (This is, in fact, also true for the other possible mechanism of rift formation downstream of an isolated grounded spot, that is, due to basal friction causing elevated downstream longitudinal stresses.) This is true of the rifts shown in Figure 4. The screenshot below shows an annotated version of this

figure showing which bits of formerly grounded ice might be associated with the rifts. We have made this argument a more explicitly in the revised manuscript.

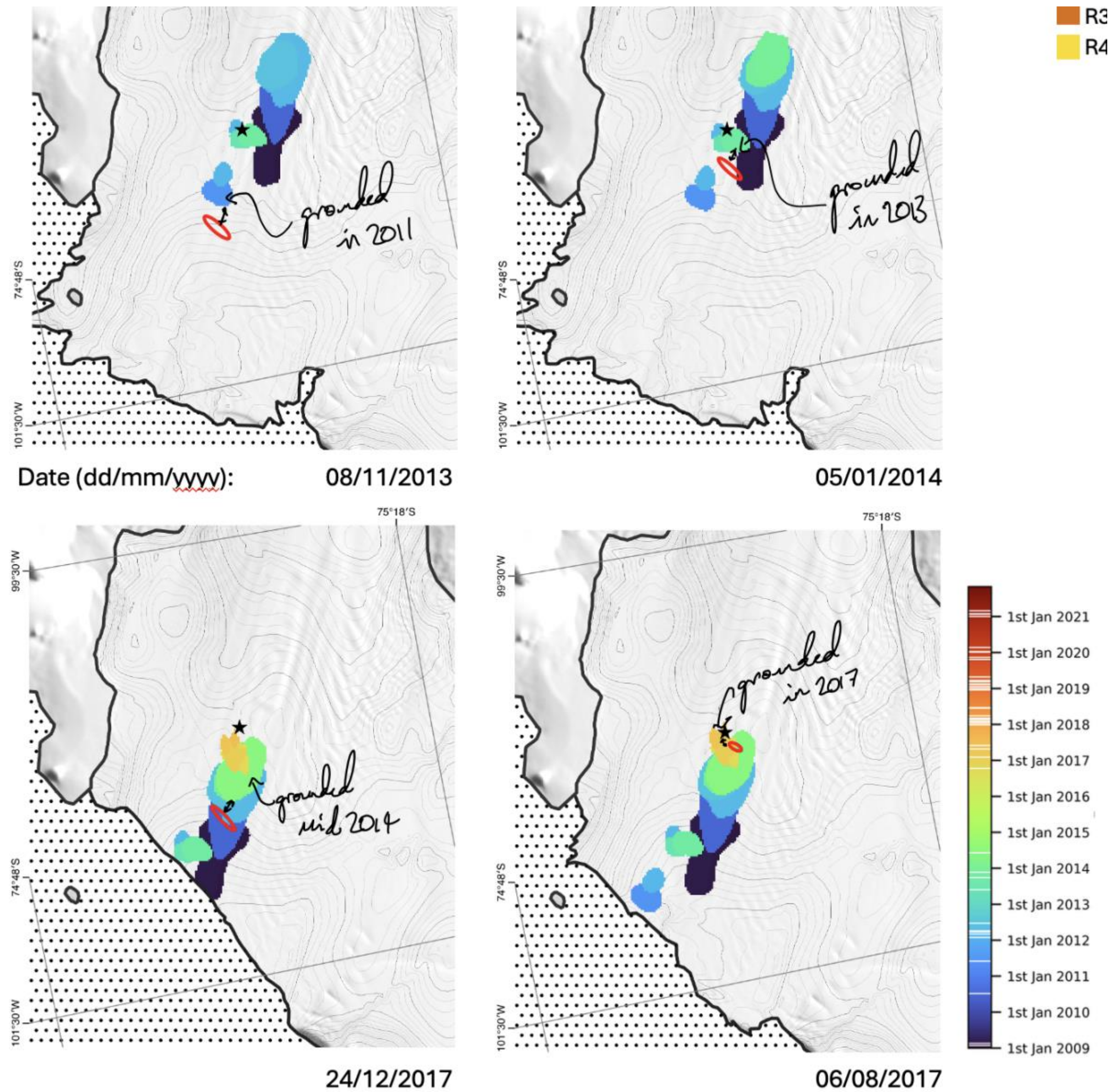

Line 164: How do you know that all surface depressions correspond to rifts or basal crevasses? I agree that what is being identified in Figure 4c is showcasing rifts, but not basal crevasses. The OIB depth radar shows clearly what are crevasses and what are rifts. It is necessary to support this interpretation with either literature or data that the surface depression are one or the other or both.

Thank you for the helpful comment and the suggestion of using IceBridge radar data. While it would be nice to distinguish rifts from basal crevasses in many contexts, it is not critical for our interpretation here—the key point is the presence of fractures. Our original wording ("rifts or basal crevasses") was intended as a catch-all, but to avoid implying a level of classification we cannot confirm, we have changed this to the less specific term "cracks." We have also clarified this in the figure caption.

Lines 171-172: Radar data shows that a rift can exist without an overlaying surface

expression. This can be observed from the CReSIS data flown over Petermann Glacier. Additionally, a surface expression will not appear until an ice shelf reaches a specific thinness. What that exact ratio is is unknown, but studies like Humbert et al, (2015) and Child et al, (2021) have shown that large bottom fractures can exist for significant periods of time before a surface expression appears.

This is a very interesting observation, thank you very much for raising it. Indeed we had this in mind when discussing the possibility that weakening of the central band of the ice shelf occurred at the grounding line. We have inserted the following sentence into the discussion: "In addition, it is well-known that basal crevasses can persist for decades before producing a clear surface expression (e.g. Humbert et al., 2015). Hence, it is plausible that the features shown in Fig. 4c originated far upstream of the bathymetric high-point, e.g. at the grounding line during a period of retreat, and became visible downstream of it only coincidentally."

Lines 172-173: The main flaw in this hypothesis is that the 2015-2020 rifts did not occur with exact timing or at equal intervals. This indicates that there were deviations in the stresses acting on the ice shelf which would not be the case with simply tidal hinging as we would expect that to behave with consistent force.

The tidal hinging can only occur when a part of the ice shelf is grounded, so it is not true that the rifts occurring at irregular intervals is evidence against this hypothesis. However, it is certainly true that there were changes in the stresses acting on the ice shelf throughout the period! We have included a paragraph at the start of the discussion of crevasses and grounding explaining how stresses have evolved on PIG.

Line 178-181: Why is an ice thickness of 500 m being assumed? Why is it not being estimated from surface elevations or airborne radar? Are any of these parameters specific directly to PIG? If not, then the 188 kPa simply shows that the stress regime COULD be such that fracturing might occur. This value is not temporally relevant and therefore could be reflective of any non-grounded deviatoric stresses.

Thank you for the comment. We agree that we could have been more specific here, and provide estimates on stress that are slightly less rough. As such, we have expanded this section to give a much more thorough account of the stresses caused by tidal motion according to an elastic beam model. We have used an estimate of thickness from the central Pine Island Ice Shelf and a timeseries of tidal amplitudes according to CATS2008.

Line 182: "elsewhere in the literature"—provide examples.

Thank you for this comment. We have provided an extensive account of the literature on the use of the elastic beam model in ice shelf flexure.

Lines 183-184: The Vaughan et al, (2012) comparison is misleading. The Vaughan et al, (2012) details a completely different fracturing scenario for fracturing that is occurring parallel to ice flow and is the result of hydrostatic adjustments causing very shallow (both surface and bottom) fracture propagations. There is no evidence provided here or in the

literature that supports that tidally-induced hinging stresses will be nearly the same as those generated from hydrostatic readjustments. None of the fractures reported in Vaughan et al, (2012) had penetrated the entirety of the ice shelf's ice thickness. I would argue that the deviatoric stresses reported in Vaughan et al, (2012) are absolutely not indicative of the intermittent burst of stress deviation required for a mid-ice-shelf-full-fracture to take place.

We agree that Vaughan et al., (2012) details a completely different fracturing scenario (though disagree that they only consider along-flow fractures), but this does not preclude us referencing it here. However, we have removed the reference as it could be misleading.

Line 187: What is the crack tip in this study's scenario? Is the implication that the rift formed from a basal crevasse? If so, that needs to be said.

The rifts could have formed from basal or surface crevasses, or a mixture of the two. The argument about recrystallisation is not dependent on the crevasse type. The same is true for laterally propagating rifts.

Line 189: Shouldn't the stresses be greater than the fracture toughness?

Thank you for the question. In this part of the article (now in the discussion), we are talking about subcritical failure where the stress is below the fracture toughness.

Line 191: This is an inappropriate reference as it pertains to freshwater ice. The most thorough studies of glacier ice fracture toughness are the Rist et al, (1999) and Rist et al, (2002) studies.

Thank you for the comment. However, we are talking about subcritical fatigue failure so do not need to reference studies on fracture toughness. We have left the current citation it in as, though different materials, freshwater ice and glacier ice are similar enough for this to be a relevant citation, for example, in both considered brittle. Instead, we have included a note to say that the referenced study is on freshwater ice.

Lines 196-197: I would argue that it is possible to have a more thorough assessment here as to why no rifts have appeared in post-2017 grounded ice shelf ice. The authors have an impressive amount of velocity data and there therefore have the ability to produce a comprehensively extensive record of the changes in strain rates and tensile stresses for the entire ice shelf. There were likely short-lived events that affected the stress regime and those events will be captured in the velocity record. Additionally, the particle tracking should be extended to when the bathymetric high grounded ice was initially flowing over PIG's ice sheet grounding line.

We thank the reviewer for their comment and suggestion that we look at strain rate data. There is nothing in the strain rate data that suggest any spikes in strain rate on the central shelf.

Lines 203-207: Both of these examples are irrelevant to this study. At no point do the authors in this study refer to the ice sheet grounding line. I fully believe that it is important to include it in a study like this, but in the current state of the manuscript, any comparisons to the ice sheet grounding line is unrelated to the present analysis. As stated earlier, the Vaughan et al, (2012) basal crevasses formed laterally—it is a completely separate stress regime than what is observed in this study.

We respectfully disagree with the idea that we should not be allowed to mention the grounding line just because we focus the study on the ice shelf. The example of Vaughan et al., (2012) is simply there to suggest that there are other ways of seeding surface and basal crevasses than tidal hinging. However, in restructuring that part of the manuscript, we anyway removed those references.

Line 275: Write out the BISICLES acronym.

It is not an acronym, just a name.

Line 277: Over what extent was the optimization problem solved for?

We have rewritten this section.

Line 296: Monthly observations of what? How do these velocities compare with those from Line 252?

Thank you for spotting that ambiguity. We have clarified these are monthly velocity observations. They are similar to those from line 252. They are more complete in terms of coverage, though are tidally-corrected which precludes them from use in the DROT method.

Line 322: Include what years the pre-2015 velocity data is from.

Thank you for the suggestion, we have included this information in the revised version on the methods.

Line 324: Why was it necessary to keep track of regions of ice that had calved?

In order that we can represent all the grounded areas on one map showing their would-be present-day locations had the ice shelf not calved.

FIGURES:
The attached pdf has my comments with regards to the paper's figures.

We thank the reviewer very much for the detailed comments on the figures. We have considered all of the suggestions and implemented them where appropriate. Below are the reviewer's comments and our responses:

Figure 1:

-There needs to be a way of affiliating each colored blob's (for lack of a better word here) origin for this figure to actually display temporal information. Give the polygon boundaries of each region's origin its own exclusive symbol (e.g., dots, astrix, use different line widths), and color-coordinate each of those boundaries with the down-flow blobs. This way the reader can visually interpretate both grounding extents and horizontal displacements.

Thank you for the suggestion. We have separated this into two sub-figures – one showing where the regrounding occurred with respect to the bedrock (a), and one showing the remapped areas as the figure used to (b). There are over fifty polygons, so we unfortunately failed to give each its own symbol and have it be readable. So the polygons are all the same style but are colour-coded by date of regrounding.

-The star symbol needs to be larger

We have made it larger.

-The grounding line needs to be a different color to the bathymetric isolines. This is especially true for the small grounded pinning point on the centrally-lower, left side of the figure.

Thank you for the suggestion. We have made the grounding line much thicker compared to the contours in bathymetry data and removed the small grounded pinning point referenced.

-Often there are depressions in bathymetry and I am wondering if that is taking place within the region that is circled in magenta. A few labels on these isolines were be helpful.

Thank you for the suggestion, we have included some more labels for these contours.

-Caption: Rather than saying "Recent grounding", provide an actual timeline.

We have implemented this suggestion.

-"Contouring shows..."— provide the provenance for the bathymetric data. "according to. Rignot et al. (2016a)"—tell the reader what the timestamp of the grounding line is.

We have implemented these suggestions.

Figure 2

-If Figure 2a is representing only grounded ice, then including the grounding line is incredibly confusing. The grounding line and the shelf terminus cannot be the same symbol.

We have changed (here and throughout) the symbol for the calving front to a dot-dashed line.

-In B, should the right y-axis be km instead of m?

Thank you for spotting this error, we have fixed it.

-Caption: "between 2016-2021"—does that mean the basal friction results are an average? "over the region shown in a"—what region is this referring to? Include what year the grounding line is supposed to be representing.

Thank you. In order: no, it does not mean basal friction results are an average; we have changed "the region in a" to "the region shown by the black dashed line in (a)"; we have now included the year the grounding line represents.

Figure 3

-Is the dashed line in the top left maps of A showing the entire length of the profile in B? It appears as though it isn't and that map needs to have its scale altered.

Thank you for the comment. We have made it clear on part (b) of the figure where the grounding line shown in part (a) is situated, which is sufficient to let the reader know what is going on.

-The grounding line and the calving front cannot have the same symbol. -In B, highlight on the x-axis where the bathymetric high is located.

We have changed the calving front to be a dot-dash line and have shown on the x-axis of part (b) where the grounding is imposed.

-Caption: Provide the year of the grounding line.

We have done so.

Figure 4

-Are the red ellipses all different rifts? If so, then they should all be a different color and that color needs to correlate to the lines in subfigure B.

Thank you for the suggestion. However, to avoid confusion with the colours used for the remapped grounded areas, we have kept them as red. We have labelled them R1-4 to make it clearer, however.

-In A, the bottom two subfigures need to be flipped with one another.

We've kept these as they were as they are ordered by the calving events caused by each rift.

-The upper two subfigures in A have an identical terminus. Is this reality? If it is, include this detail in the caption. If it is not, then include the appropriate terminus boundaries for each subfigure.

This is not reality, though it is close-to and makes little difference to the point we are making with the figure, so we have not made this change.

-For A, make the grounding line and the terminus positions different symbols.

Thank you for this suggestion. We have implemented this change.

-In B, do those colors have any affiliation with the timeline colors in A? If not, then the colors in B need to be different to those in A. If there is an affiliation between the timeline colors and rift colors, then be sure to tell the reader that in the caption.

There is no affiliation between the colors in A and B, but the separate colourbars should make this clear.

-Like my comment in Figure 1, then black dashed-lined polygons are incredibly confusing to interpret. These colors need to be reflective of timeline origin color bar.

Could the reviewer clarify what this is referring to? For example, what is the "timeline origin colour bar"?

-In B, next to each rift, include the month/year of initial observation.

Thank you for the suggestion, but this information is in (a).

-In C, what exactly are the arrows highlighting? Is it simply surface expressions of rifts and basal crevasses in 2014? Or are they highlighting the locations of the rifts from B? If it showing a 2014 timestamp of surface undulations, this is the sort of information that should be in a Supplemental and not in a main figure.

The arrows are highlighting surface undulations indicate rifts or deep basal crevasses in 2014. Thank you for the suggestion, but we disagree that this should be a supplementary figure.

-For C, it would be more impactful to show whether any of the initial observations of rifts correlates to a specific “blob” of advected ice. This way the authors could hypothesize whether any of the bathymetric high grounding events are likely the driver of initial fracture opening.

Thank you, though this is what part (a) of the figure is supposed to do.

-Caption: “indications”—awkward choice of word for “depression” or “undulation”. “wake of the region”—advected down-flow? “found as the union of grounded spots we identified and advected with flow”—I do not understand what is being communicated here. “the background image for each”—reaplce “each” with “a and b”.

Thank you for raising this confusion. We have rewritten the figure caption to make things clearer.

REFERENCES

Child, Sarah F., Leigh A. Stearns, C. J. van der Veen, and Pedro Elosegui. "Basal crevasse formation on Byrd glacier, East Antarctica, as proxy for past subglacial flooding events." Geophysical Research Letters 48, no. 15 (2021): e2020GL090978.

Cornford, Stephen L., Daniel F. Martin, Daniel T. Graves, Douglas F. Ranken, Anne M. Le Brocq, Rupert M. Gladstone, Antony J. Payne, Esmond G. Ng, and William H. Lipscomb. "Adaptive mesh, finite volume modeling of marine ice sheets." Journal of Computational Physics 232, no. 1 (2013): 529-549.

Cornford, Stephen Leslie, D. F. Martin, A. J. Payne, E. G. Ng, A. M. Le Brocq, Rupert M. Gladstone, Thomas L. Edwards et al. "Century-scale simulations of the response of the West Antarctic Ice Sheet to a warming climate." The Cryosphere 9, no. 4 (2015): 1579-1600.

Davison, Benjamin J., Anna E. Hogg, Noel Gourmelen, Livia Jakob, Jan Wuite, Thomas Nagler, Chad A. Greene, Julia Andreasen, and Marcus E. Engdahl. "Annual mass budget of Antarctic ice shelves from 1997 to 2021." Science Advances 9, no. 41 (2023): eadi0186.

Humbert, Angelika, Daniel Steinhage, Veit Helm, Sebastian Hoerz, Jacqueline Berendt, Elke Leipprand, Julia Christmann, Carolin Plate, and Ralf Müller. "On the link between surface and basal structures of the Jelbart Ice Shelf, Antarctica." Journal of Glaciology 61, no. 229 (2015): 975-986.

Joughin, Ian, David E. Shean, Ben E. Smith, and P. Dutrieux. "Grounding line variability and subglacial lake drainage on Pine Island Glacier, Antarctica." Geophysical Research Letters 43, no. 17 (2016): 9093-9102.

MacAyeal, Douglas R. "A tutorial on the use of control methods in ice-sheet modeling." Journal of Glaciology 39, no. 131 (1993): 91-98.

Mouginot, J., E. Rignot, and B. Scheuchl. "Sustained increase in ice discharge from the Amundsen Sea Embayment, West Antarctica, from 1973 to 2013." Geophysical Research Letters 41, no. 5 (2014): 1576-1584.

Rist, M. A., P. R. Sammonds, S. A. F. Murrell, P. G. Meredith, C. S. M. Doake, Hans Oerter, and K. Matsuki. "Experimental and theoretical fracture mechanics applied to Antarctic ice fracture and surface crevassing." Journal of Geophysical Research: Solid Earth 104, no. B2 (1999): 2973-2987.

Rist, M. A., P. R. Sammonds, Hans Oerter, and C. S. M. Doake. "Fracture of Antarctic shelf ice." Journal of Geophysical Research: Solid Earth 107, no. B1 (2002): ECV-2.

Schoof, Christian, and Richard CA Hindmarsh. "Thin-film flows with wall slip: an asymptotic analysis of higher order glacier flow models." Quarterly journal of mechanics and applied mathematics 63, no. 1 (2010): 73-114.

Van Der Veen, Cornelis J., and I. M. Whillans. "Force budget: I. Theory and numerical methods." Journal of Glaciology 35, no. 119 (1989): 53-60.

Vaughan, David G., Hugh FJ Corr, Robert A. Bindschadler, Pierre Dutrieux, G. Hilmar Gudmundsson, Adrian Jenkins, Thomas Newman, Patricia Vornberger, and Duncan J. Wingham. "Subglacial melt channels and fracture in the floating part of Pine Island Glacier, Antarctica." Journal of Geophysical Research: Earth Surface 117, no. F3 (2012).

Responses to second round of reviewer comments on "Impact of two decades of intermittent ice shelf regrounding on the dynamics of Pine Island Glacier, Antarctica"

We would like to thank the reviewers for the time they have spent looking over this article. The feedback they have provided is well-considered and valuable, and, we hope they agree, has led to a much-improved manuscript.

This document reproduces both reviews in full along with our responses. The great majority of the time, we have implemented the suggestion of the reviewer and, where we haven't, have provided justification.

Overall, we have not made any large structural changes to the manuscript or changes to the overall message. Instead, we have incorporated comments and suggestions by inserting small amounts of text and making minor edits to figures throughout.

Below are each reviewer's comments and our responses. Our responses are coloured in blue.

Reviewer #3 (Remarks to the Author):

I thank the authors for the changes to the manuscript that have improved it and agree that the revised manuscript is improved. After reading their responses and the new manuscripts, I have only some minor remaining concerns that need to be addressed. Thanks for the additional explanation and changes to the text regarding tidal hinging promoting calving. I am supportive of the reframing of the conclusions that the primary conclusion (shown by the experiments in the manuscript) relate the lack of buttressing provided by the regrounding. Although the tidal hinging in the paper presented is a possible explanation for the fracture initiation and growth, the major weakness of this study is its inability to directly link the ice sheet model with the predicted calving. The redrafting of the inverse methods has substantially improved the manuscript. I now more fully appreciate the authors methods which I am supportive of. I have a couple of minor points I think would improve the manuscript.

We thank the reviewer very much for the general vote of confidence in the manuscript. We agree that the inability to link the model with predictions of calving is a weakness, but think that this will have to wait for future work.

• The assumption that the entire ice shelf is grounded in the first set of inverse experiments is stated in the main text, but it would be useful if it was very briefly noted in section 6.2 too.

Thank you for noticing this omission. We have added the line "…in which we pretend that the ice shelf is fully grounded" to the first sentence of section 6.2.

• The distinction with the diagnostic simulations is much clearer now. Ln 325-326, "This inverse problem is distinct from those discussed in Sec. 6.2 …" should be altered as these results are presented in the main text rather than purely for initialisation. Perhaps instead state that these experiments are conducted for a fixed timeframe. I thank the authors for their explanation of how much they had to change the basal geometry to induce regrounding. It needs to be stated how uncertain the bathymetry is and by how much it was elevated. I don't see it as a weakness that it had to be adjusted so much if the data is so uncertain. This can probably be stated in the methods if the authors are uncomfortable with the impression this gives. However, justification for this (reasonable) method needs to be stated in the main text. The need for accurate bathymetry/bed geometry to model calving/basal stress is well known.

We don't think we do present these results in the main text, so have left this sentence in just to make it clear that we are not interested in the actual solutions to this particular inverse problem, only to the set of inverse problems in which the ice shelf is fully grounded.
Thank you for the suggestion of adding some more explanation on how we raised the height of the bedrock. We have added to the methods the lines: "The bedrock over this region had to be raised by an average of 150m. This suggests a significant underestimation of bedrock elevation in the region, as can be seen in Fig. 1 b (white star),

which shows grounding to have most frequently occurred on what the bedrock data suggest is a local bathymetric low point." I have also added: "The artificial grounding was done by locally raising the height of the subshelf bathymetry over the small region we wanted to ground." to section 3 of the main text.

Overall, I think this paper makes a useful contribution to our understanding of the role buttressing plays in ice shelf dynamics and a potential source of fraction initiation. I would encourage the authors to further consider how such processes can be incorporated in icesheet models in the future.

We thank the reviewer again for their time reviewing this article, and promise to try!

Minor comments

Ln 107 '(they are all ficticious)'. This takes away from the fact that these experiments are reasonable and justifiable. Additionally, it would be useful to note here why they are notional values. I suggest something like, "which are notional/theoretical as grounding is imposed on the entire ice shelf. Instead, we focus...". Note fictitious is misspelt.

Thank you for the comment and suggestion, we have changed this to "...which are only notional given that the whole ice shelf is artificially grounded..."

Ln 128-130. Should these not refer to Fig 3 and not to Fig. 2?

Thank you for spotting this mistake – it has been rectified.

Ln 148-154. It is not clear what 'this' at the start of each sentence is referring to for all four out of the five sentences in the paragraph. Please rewrite for clarity.

Apologies for this – it is a habit I find difficult to break. We have rewritten the paragraph to make it clearer what each use of "this" is referring to. Thank you for your patience.

Ln 196-198. Again with neighbouring sentences it is unclear what 'this' is referring to.

We have altered a few sentences in this paragraph to make it more clear what is being referred to throughout.

Ln 231. Change 'this' to 'regrounding' or' grounding'.

Thank you for the suggestion. We have implemented this change.

Reviewer #4 (Remarks to the Author):

This study deals with the analysis of the impact of intermittently grounding of the Pine Island ice shelf on high points in the subshelf bedrock over the last two decades. A noteworthy conclusion is that regrounding provided very little resistance to the flow of Pine Island Ice Shelf. Regrounding happened during a period of glacier speed-up and terminus retreat. Based on the results, regrounding may even have played a role in terminus retreat.

We thank the reviewer for their clear account of the argument presented.

General comments:

1) Remote sensing part:

DROT vs DINSAR: A stronger case for the use of DROT over DINSAR would be warranted. As written, the authors open themselves up to the question why they mention DINSAR, highlight its greater sensitivity, yet do not test the method. This particularly in light of the fact that by calculating offsets, 80% of the work for DINSAR is already done.

Thank you for the thoughtful comment. We considered the use of D-InSAR but opted for DROT as our focus was on capturing as many periods of grounding as possible. As the reviewer correctly says, we acknowledge that D-InSAR is in some senses more sensitive. However, that is only the case when there is a high level of coherence between image acquisitions. DROT requires some level of coherence, but much less. Because snowfall and melt are common on Pine Island ice shelf, DROT is a more reliable method for our purposes. We have added to the methods the following sentences to make this clearer to the reader: "We chose to use DROT rather than D-InSAR for this study as we prioritised dense temporal coverage in our observations of regrounding. DROT requires a lower level of interferometric coherence between image acquisitions, which is sometimes limited in this part of Antarctica due to snow deposition and changes in surface moisture."

Crack monitoring and visibility: Clarify what imagery was used to monitor crack propagation and specify the resolution. Cracks may have formed and weakened the ice well before they are visible in imagery. Is there any impact of mixing SAR and optical SAR and optical data? Were the cracks manually outlined, what was your detection threshold? Does SAR image geometry play a role for visibility?

Thank you for the questions. We have added the resolutions of the Sentinel-1, Sentinel-2 and Landsat-8 imagery to the manuscript. Respectively, they are: 5 × 20 m posted to a 10×10 m grid, 10×10 m and 30×30 m. Mixing SAR and optical data is useful for getting the greatest temporal coverage. Both have their strengths. Given that viewing geometry within the same acquisition frame is the same, SAR is most consistent way to measure change, while optical data depends more on solar illumination angle and cloud cover. SAR allows imaging during the winter which is useful, some penetration through snowbridges and some imaging of sub-resolution features such as crevasse walls which

are strong reflectors. The visibility of features in optical imagery depends much more on the surface slope, so is useful for locating surface depressions due to basal crevasses. Cracks were indeed manually delineated and there was no strict detection threshold beyond being able to see the crevasse in the imagery. As always, SAR image geometry plays a role in visibility. The Amundsen Sea sector is both imaged from a few different angles, and covered by timeseries of images acquired using the same imaging geometry.

2) Modeling part:

The primary concern relates to the experiment in which the entire ice shelf is artificially grounded. It is unclear why the authors expect spatial variability in basal drag under these conditions. If the ice shelf is not in contact with the seamount, the velocity field across the shelf should remain relatively smooth, and substantial spatial variations in basal drag would seem unlikely.

In addition, while the authors report changes in basal drag (in kPa), they do not quantify the corresponding reduction in velocity. Although presenting basal drag alone is acceptable, the omission of velocity changes limits the interpretation of the results. Notably, they report a 5% reduction in speed when grounding only a small portion of the ice shelf. This raises the question of what impact fully grounding the ice shelf has in the first experiment, and why the associated velocity changes were not included.

Thank you for the questions.
On the first question: For the experiment in which the entire ice shelf is artificially grounded, we used a set of velocity observations covering a period (2016-2021) during which the ice shelf was often in contact with the seamount. In that case, we might expect the velocity data to contain some information pointing towards localised basal traction. Because membrane stresses are transferred many ice thicknesses, it is not certain that evidence for localised basal traction would be immediately visible to the eye as a localised feature in the ice velocity. Hence the idea of using the inverse problems as an additional check. It's reasonable to say that our results are not unexpected, but we are not of the opinion that they were inevitable.
On the second question: In the first experiment, the model is able to fit the velocity fairly well (even with much larger areas of nonzero basal friction than provided in the second experiment) by reducing the viscosity in the shear margins. We note this in the manuscript. We don't report changes in modelled velocity as we are fitting it to observations, so it wouldn't be very useful.

Specific comments:

Line 23: 'the late 2000s', so does this mean that the retreat started in the 70s? This sentence is not very clear.

Thank you for the comment, this could have been a lot clearer. We have removed mention of the 1970s as it was confusing matters. The sentence now reads “The early stages of retreat from this ridge are difficult to characterise due to a lack of observations; however, satellite records show that by the 1990s retreat was ongoing and lasted until the late 2000s”.

Line 25: 'available intermediate steady-state ...' What does it mean? What do you mean with steady-state? Isn't this because there are no observations during that period?

This wasn’t particularly clear, thank you for raising it. We have altered this to now read “... lack of available stable grounding line positions.”

Line 31: 'during the 2000s'. Is this shown in Figure 1?

It’s a little unclear when exactly this separation occurred. It’s discussed in the reference to Joughin, 2016 given in the article, but we don’t make reference to figure 1.

Line 56: what does 'positive' perturbations mean?

Thank you for the comment. We mean perturbations that are positive in sign – i.e. increases in thickness.

Line 67: "...combination of TerraSAR-X and Sentinel-1 data." Clarify that the data was processed separately

Thank you for the comment. We have changed “in our case, a combination of TerraSAR-X and Sentinel-1 data” to “in our case, TerraSAR-X or Sentinel-1 data” so it doesn’t seem like we combined the two for individual DROT retrievals.

Line 75: 'in the 1980s'. Can you provide a reference for this claim?

Thank you for noting this omission. We have included a reference to Jenkins et al., 2010 here, as in the introduction.

Line 80: Why would thinning of the ice shelf and passage of a thick ice parcel over the bathymetric high point have the same impact, shrinking of the grounded area

Apologies, that isn’t particularly clear – it’s a tricky bit to word. We meant the advection of thick ice from the location of bathymetric high-point to further downstream. We have adjusted this wording to read: “... or an end to the advection of a thick parcel of ice over the bathymetric high-point”.

Line 88: 'Thus one could imagine...' I don't understand this sentence. In the sentence before, you say that you are not 'confident' about past regrounding. Does this mean that ice would be progressively grounded over a deeper bed if we could go back in time and observe it?

The sentence before says we are not confident that our first observation indicates the start of the regrounding. As stated in the next sentence, there is evidence of regrounding near the bathymetric high-point in 1999 from D-InSAR. However, as discussed above, D-InSAR isn't a very good tool for determining how much grounding there has been. The sentence in question says that the fact that we don't see much prior to 2009 does not mean that regrounding didn't regularly occur before then – we just don't have the observations to know for sure.

Line 94: Sentinel-1 shortest repeat period. Recommend to mention 6 days.

Thank you for the suggestion, we have added a note that this period is 6 days

Line 102: I am not sure I understand the need for saying 'each month'. Does this mean that the ice-shelf is grounded during the period 2016-2021?

That's a very good point, thank you for raising it. We have combined this with the next sentence so it's clear that the "for each month" refers to the whole inverse problem, rather than just changing the bedrock elevation to ground the ice shelf.

Line 107: please define stickiness. Despite understanding what the authors refer to (coefficient C, also mentioned in the methodology), I don't think this is the correct terminology.

We have changed this to "basal traction" to be less ambiguous.

Line 110: What is the % impact on glacier velocity based on artificially grounding the ice shelf with 4kPa of basal drag? Later in this section, you quantify the slowdown due to partially grounding the ice shelf, but the same analysis for the first experiment is missing.

This is a good point, thank you for raising it. The 4kPa of additional basal drag across the whole shelf doesn't change the speed much (as can be seen in Figure 2b). This is because the model was able to fit the speed relatively well, despite not reducing basal traction to zero under the floating ice, by reducing the viscosity of the shear margins. We make this statement on line 115 of the new version of the manuscript.

Line 111: 'For context ...' Do you have a reference for the 'representative stress scale'?

We don't have a reference for that, but if you compute the driving stress at the calving front it comes out at ~100 kPa. This is very typical for ice streams in Antarctica.

Line 113: Couple of hundred meters: Very casual formulation.

We have changed this to "around 200 m".

Line 115: 'While the recovered ...' I don't understand why you would expect some

spatial variability in the inferred friction coefficient, given that the velocity map is relatively smooth over the ice shelf.

This is fair enough, though this conclusion was not obvious to us a-priori and we think it won't be obvious to lots of readers. It's not unreasonable to think there might be features in the velocity data which reflect regions of basal traction that are not immediately visible to the eye – this comes up regularly in solutions to inverse problems.

Line 195: this paragraph is confusing. Please see my comments below.

Thank you for your comments. We have adjusted this paragraph and hope it is clearer.

Line 196: 'This suggests ...' this sentence is somewhat confusing. What is the 'hydrological' connection between a weekly deformable till and 'low effective pressure'. I may have missed this, and I apologize if I did, but this should be made more clear for the reader.

Thank you for the comment. We have rephrased it slightly so that it's clear a weakly deformable till can occur without a strong hydrological connection to the ocean.

Line 199: 'unwise'? I don't understand this.

We have added "as this could lead to over-estimation of basal traction".

Line 200: 'the merits of effective-pressure-dependent sliding laws'. I am not sure I understand. In section 3, you say that grounding the ice shelf (using which friction law?) does not change velocity over the grounded glacier. If that is the case, I am not sure you can claim the 'merits' of this type of friction laws from the results shown here. I do agree on the importance of including effective pressure, but this goes beyond the scope of this manuscript. Again, I may have misinterpreted this section, and I apologize if I did. I would appreciate some clarification.

These are all good questions! On the first point, a regularised Coulomb formulation (equation 4) is used when we need a sliding law. (For the inverse problems presented in figure 2, we are just interested in the stress so the form of the sliding law doesn't matter much.) The point of this sentence is just to suggest that including effective pressure in the sliding formulation might help to avoid overestimating the basal traction felt by ice that regrounds on the sea floor. We have added a note to this effect at the end of the paragraph.

Line 240: Zenodo link is missing.

Thank you. We will incude the zenodo link when the article goes to publication. All the files we will upload were included as material for reviewers.

Line 280: What friction law do you use to compute velocity changes?

For the inverse problems, we use a linear sliding law and for the simulations in which we add traction to a small region on the ice shelf, we use a regularised Coulomb law. These are both mentioned in the methods section of the manuscript.

Figures:

Figures 1,3,4: please consider using a colorblind-friendly colorbar that is different from jet.

Thank you for raising this good point. Before settling on the colourmaps to use in the figures for this manuscript, we checked their colourblind friendliness using the website https://www.color-blindness.com/coblis-color-blindness-simulator/. None of the colourmaps are jet, but something we thought would be more suitable.

Figs 3 & 4: Suggest that every panel gets a letter.

Thank you for the suggestion, we have added letters to each panel of figure 4. However, we have kept figure 3 the same and hope that the labels indicating the basal stress differentiate the panels clearly enough.

Figure 4: it is hard to see the bathymetry contour.

We have made a higher-resolution figure where the contour is more visible – thank you for raising this.

Reviewer #5 (Remarks to the Author):

I co-reviewed this manuscript with one of the reviewers who provided the listed reports. This is part of the Nature Communications initiative to facilitate training in peer review and to provide appropriate recognition for Early Career Researchers who co-review manuscripts.

Excellent!

FIGURES
Note for all figures, they need scale bars.

Figure 1:
-There needs to be a way of affiliating each colored blob's (for lack of a better word here) origin for this figure to actually display temporal information. Give the polygon boundaries of each region's origin its own exclusive symbol (e.g., dots, astrix, use different line widths), and color-coordinate each of those boundaries with the down-flow blobs. This way the reader can visually interpretate both grounding extents and horizontal displacements.
-The star symbol needs to be larger
-The grounding line needs to be a different color to the bathymetric isolines. This is especially true for the small grounded pinning point on the centrally-lower, left side of the figure.
-Often there are depressions in bathymetry and I am wondering if that is taking place within the region that is circled in magenta:

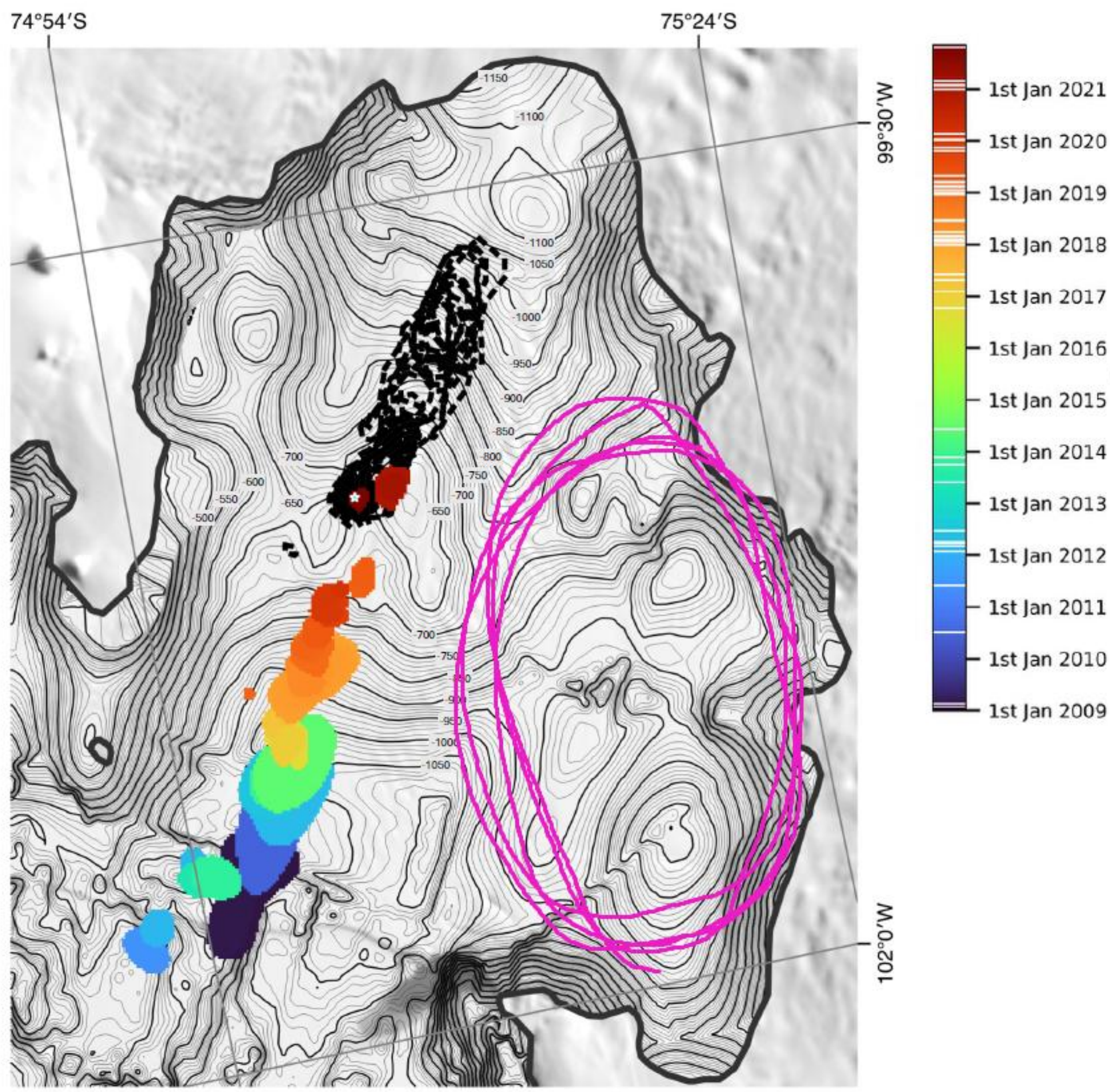

A few labels on these isolines were be helpful.

-Caption: Rather than saying “Recent grounding”, provide an actual timeline. “Contouring shows…”—provide the provenance for the bathymetric data. “according to. Rignot et al. (2016a)”—tell the reader what the timestamp of the grounding line is.

Figure 2
-If Figure 2a is representing only grounded ice, then including the grounding line is incredibly confusing. The grounding line and the shelf terminus cannot be the same symbol.

-In B, should the right y-axis be km instead of m?

-Caption: “between 2016-2021”—does that mean the basal friction results are an average? “over the region shown in a”—what region is this referring to? Include what year the grounding line is supposed to be representing.

Figure 3
-Is the dashed line in the top left maps of A showing the entire length of the profile in B? It appears as though it isn’t and that map needs to have its scale altered.

-The grounding line and the calving front cannot have the same symbol.

-In B, highlight on the x-axis where the bathymetric high is located.

-Caption: Provide the year of the grounding line.

Figure 4
This is a very confusing figure.

-Are the red ellipses all different rifts? If so, then they should all be a different color and that color needs to correlate to the lines in subfigure B.

-In A, the bottom two subfigures need to be flipped with one another.

-The upper two subfigures in A have an identical terminus. Is this reality? If it is, include this detail in the caption. If it is not, then include the appropriate terminus boundaries for each subfigure.

-For A, make the grounding line and the terminus positions different symbols.

-In B, do those colors have any affiliation with the timeline colors in A? If not, then the colors in B need to be different to those in A. If there is an affiliation between the timeline colors and rift colors, then be sure to tell the reader that in the caption.

-Like my comment in Figure 1, then black dashed-lined polygons are incredibly confusing to interpret. These colors need to be reflective of timeline origin color bar.

-In B, next to each rift, include the month/year of initial observation.

-In C, what exactly are the arrows highlighting? Is it simply surface expressions of rifts and basal crevasses in 2014? Or are they highlighting the locations of the rifts from B? If it showing a 2014 timestamp of surface undulations, this is the sort of information that should be in a Supplemental and not in a main figure.

-For C, it would be more impactful to show whether any of the initial observations of rifts correlates to a specific “blob” of advected ice. This way the authors could hypothesize whether any of the bathymetric high grounding events are likely the driver of initial fracture opening.

-Caption: “indications”—awkward choice of word for “depression” or “undulation”. “wake of the region”—advected down-flow? “found as the union of grounded spots we identified and advected with flow”—I do not understand what is being communicated here. “the background image for each”—reaplce “each” with “a and b”.
